# The Bactofilin Cytoskeleton Protein BacM of *Myxococcus xanthus* Forms an Extended β-Sheet Structure Likely Mediated by Hydrophobic Interactions

**DOI:** 10.1371/journal.pone.0121074

**Published:** 2015-03-24

**Authors:** David M. Zuckerman, Lauren E. Boucher, Kefang Xie, Harald Engelhardt, Jürgen Bosch, Egbert Hoiczyk

**Affiliations:** 1 W. Harry Feinstone Department of Molecular Microbiology and Immunology, Johns Hopkins Bloomberg School of Public Health, Baltimore, Maryland, United States of America; 2 Department of Biochemistry and Molecular Biology, Johns Hopkins Bloomberg School of Public Health and Johns Hopkins Malaria Research Institute, Baltimore, Maryland, United States of America; 3 Department of Structural Biology, Max Planck Institute of Biochemistry, Am Klopferspitz 18, Martinsried, Germany; University of Iowa, UNITED STATES

## Abstract

Bactofilins are novel cytoskeleton proteins that are widespread in Gram-negative bacteria. *Myxococcus xanthus*, an important predatory soil bacterium, possesses four bactofilins of which one, BacM (Mxan_7475) plays an important role in cell shape maintenance. Electron and fluorescence light microscopy, as well as studies using over-expressed, purified BacM, indicate that this protein polymerizes *in vivo* and *in vitro* into ~3 nm wide filaments that further associate into higher ordered fibers of about 10 nm. Here we use a multipronged approach combining secondary structure determination, molecular modeling, biochemistry, and genetics to identify and characterize critical molecular elements that enable BacM to polymerize. Our results indicate that the bactofilin-determining domain DUF583 folds into an extended β-sheet structure, and we hypothesize a left-handed β-helix with polymerization into 3 nm filaments primarily *via* patches of hydrophobic amino acid residues. These patches form the interface allowing head-to-tail polymerization during filament formation. Biochemical analyses of these processes show that folding and polymerization occur across a wide variety of conditions and even in the presence of chaotropic agents such as one molar urea. Together, these data suggest that bactofilins are comprised of a structure unique to cytoskeleton proteins, which enables robust polymerization.

## Introduction

All living cells organize their cytoplasm to ensure efficient growth and cell division. This organization relies in part on cytoskeleton proteins, versatile scaffolds that are crucial for essential processes ranging from cell shape maintenance to morphogenesis, polarity determination, cell growth and division, chromosomal segregation, and transport of cellular cargoes (for reviews see [1–8]). While research has focused for decades on the eukaryotic cytoskeleton proteins actin, tubulin, and intermediate filaments [9–11], it has only recently been realized that bacteria possess structural homologues of these proteins, as well as a number of uniquely bacterial cytoskeletal proteins thus far not described for eukaryotes [12,13].

While sequence homology searches initially failed to identify prokaryotic homologues to eukaryotic cytoskeletal proteins, determination of the structure of the cell-division protein FtsZ revealed that it is a close structural homologue to eukaryotic tubulin, showing particularly high similarity in the GTP-binding domain [5,14]. In contrast, the bacterial actin protein MreB was initially identified based on sequence homology mapped to the eukaryotic actin ATPase domain [15] and later confirmed as an actin-like cytoskeletal protein through sequence homology in regions responsible for actin’s overall structure [16]. Outside of these regions, MreB lacks sequence homology to actin, but was found to possess a strikingly similar tertiary structure [17]. Like its many homologues and paralogs, MreB controls the cellular morphology of non-spherical bacteria. Finally, bacterial intermediate filament-like proteins were identified in bacteria with the discovery of crescentin in *Caulobacter crescentus* [18]. Like their eukaryotic counterparts, bacterial intermediate filament (IF) assembly is non-polar *via* long coiled-coil domains, structural elements that are found in many bacterial proteins, indicating that IF-like cytoskeletons are potentially widespread in bacteria [19–23]. Together, these discoveries underscore the necessity of determining the structure of cytoskeletal proteins, as this helps not only to understand the assembly and function of these structures, but also to identify evolutionarily distant homologues and relatives. This is particularly true for cytoskeleton protein families that are found uniquely in bacteria and lack obvious eukaryotic counterparts. One such uniquely bacterial cytoskeletal system is the Walker A cytoskeletal ATPases (WACAs), a family of proteins defined by their ATP-binding domain [5]. The best-studied example of a WACA is the ParA protein, which forms a rudimentary mitotic apparatus that partitions chromosomes or plasmids into daughter cells during cell division [24].

Another recently discovered family of uniquely bacterial cytoskeletal proteins is the bactofilins (reviewed in [13]). Bactofilins are small proteins, nearly ubiquitous in Gram-negative bacteria, which are defined by the presence of the highly conserved domain DUF583 [25]. The first member of this family to be identified, the protein CcmA of *Proteus mirabilis*, was discovered in a transposon-based genetic screen [26]. Insertion of the transposon at the C-terminal region of the ORF led to expression of a truncated CcmA, and resulted in mechanically fragile cells with severe morphological deformations. Later, homologues of CcmA were discovered in *C*. *crescentus*, *Helicobacter pylori*, *and M*. *xanthus* and, in all cases, found to be important for cell shape maintenance [27–29]. Deletion of the bactofilin gene *bacA* in *C*. *crescentus* led to a reduction in stalk length, while overexpression of either *bacA* or *bacB* fused to a large fluorescent protein caused an increase in the curvature of the cell [27]. Similarly, deletion of the bactofilin gene *ccmA* in *H*. *pylori* resulted in the loss of the characteristic helical cell shape, dramatically straightening the cells [28]. Finally, lack of BacM in the normally rod-shaped *M*. *xanthus* produced aberrant morphologies ranging from mildly bent to severely crooked cells [29]. The cytoskeletal nature of bactofilins was further confirmed by immunofluorescence microscopy, which revealed that BacM has a filament-like staining pattern in *M*. *xanthus*, and, when biochemically isolated, is recovered as bundle-forming fiber [29]. Similarly, when *bacA* from *C*. *crescentus* or the *M*. *xanthus* bactofilins were exogenously over-expressed in *E*. *coli*, filamentous forms of the proteins were recovered, indicating that these proteins have a high propensity to polymerize [27,29]. Based on bioinformatics [28], protein-protein interaction studies [27], and increased sensitivity of bactofilin mutants to cell-wall targeting enzymes [29], it appears that bactofilins may exert their influence over the cell morphology by contributing to proper peptidoglycan (PG) maintenance. In addition to morphology, bactofilins may affect other cellular functions. In a recent report, a BacM paralog in *M*. *xanthus*, BacP, was found to recruit and localize SofG, a small GTPase important to cell motility, to the cell pole [30]. Additionally, an mCherry fusion of SO1662, the only bactofilin identified in *Shewanella oneidensis*, assembled as a fluorescent band at midcell suggesting a possible involvement in cell division [27]. Thus, bactofilins may be versatile scaffolds that recruit and localize enzymes (i.e. PG synthesis and remodeling enzymes) and structural proteins (i.e. components of the type IV pilus and cell division machineries) to specific cellular locations.

Other than electron microscopic analyses of negatively stained, purified filaments, no other structural information has been reported for any of the bactofilins [27,29]. Since these proteins do not share sequence homology to other structurally analyzed cytoskeletal or filament-forming proteins and have not yet been subjected to further ultrastructural analysis, no data exist about the molecular basis for their ability to polymerize. Detailed analyses of their amino acid sequences suggest a three-domain structure in which N- and C-terminal domains with broad sequence variability flank the highly conserved bactofilin domain DUF583. The N-terminus of bactofilins from several species appears to be used to anchor the protein to membranes [26,29], and appears to play no role in polymerization [29]. No information has yet been reported for the function of the C-terminus, although one plausible idea is that this part of the protein is involved in the recruitment of interaction partners. Moreover, given the extreme variability of the amino acid sequences of the N- and C-termini it can currently not be ruled out that for some bactofilins, the roles of the two termini are reversed.

In this study, we express the bactofilin domain and C-terminus of BacM and find that polymers form spontaneously in the absence of nucleotides under a wide variety of conditions. To determine the basis of this polymerization, we sought to analyze the structural features of BacM. Circular dichroism and infrared spectroscopic analysis of the protein indicate that the bactofilin domain contains no α-helices, but an extended β-sheet. Bioinformatic analysis of bactofilin homologs reveals a repeat region containing highly conserved glycines and hydrophobic residues. We use these data to evaluate *in silico* 3D models of the bactofilin domain of BacM and predict a left-handed β-helix-like fold, a structural motif not previously demonstrated for any bacterial cytoskeleton proteins. Using this *in silico* model, we examine potential protein-protein interactions that would result in the extended β-sheet structure we measured, and could result in spontaneous filament formation. We hypothesize that homo-dimer interactions occur *via* head-to-tail stacking of individual subunits. Systematic mutations of predicted important amino acids abrogate the function of the protein *in vivo* and disrupt filament formation *in vitro*, in support of the model. In summary, these results enhance our understanding of this important family of cytoskeletal proteins and pave the way towards assigning individual domains and amino acids to specific functional and structural aspects of the protein.

## Materials and Methods

### Bacterial Strains and Culture Conditions

All *M*. *xanthus* strains used in this study are listed in Table 1 and are derived from the wild type strain DK1622 [31]. Cells were grown either on CTT medium (1% casitone, 10 mM Tris-HCl pH 8.0, 8 mM MgSO_4_, 1 mM KH_2_PO_4_) or CTT agar (CTT medium solidified with 1.5% agar) containing 15 μg/ml oxytetracyclin when necessary. *Escherichia coli* strains were cultured in LB medium or on LB agar plates supplemented with 100 μg/ml ampicillin or 20 μg/ml tetracycline when necessary.

**Table 1:** Bacterial strains used in this study.

Strain	Relevant description	Source or reference
*M*. *xanthus*		
DK1622	Wild type	[31]
EH301	Δ*bacM*	[29]
EH344	[EH301] P_*oar*_:*bacM*. Complementation at the chromosomal *attB* site under control of the *oar* promoter. Tet^R^.	[29]
EH106	[EH301] P_*oar*_:*bacM* Δ*C-term*. Complementation at the chromosomal *attB* site under the control of the *oar* promoter. Tet^R^.	This study
EH171	[EH301] P_*oar*_:*bacM* I124D/F125R. Complementation at the chromosomal *attB* site under control of the *oar* promoter. Tet^R^.	This study
EH175	[EH301] P_*oar*_:*bacM* L35E. Complementation at the chromosomal *attB* site under control of the *oar* promoter. Tet^R^.	This study
***E*. *coli***		
BL 21 Star (DE3)	*E*. *coli* host for protein expression.	Invitrogen
TOP10	*E*. *coli* host for plasmid maintenance.	Invitrogen

### Plasmid and Strain Construction

All strain constructions were performed as previously described [29]. To generate *M*. *xanthus* strains possessing mutant versions of BacM, plasmid *pMKK224* containing the *bacM* gene under the control of the *Oar* promoter [29] was used as a template for site-directed mutagenesis. Pfu turbo (Stratagene, LaJolla, CA) together with appropriate primers (Table 2) introduced the desired mutations, which were confirmed by sequencing of the recovered plasmids. The plasmids were then introduced into an *M*. *xanthus* Δ*bacM* strain (EH301) *via* electroporation, and integration into the chromosome was selected by resistance to oxytetracycline. Expression of the mutated versions of BacM was confirmed by immunoblotting with an anti-BacM antibody as described [29]. For expression in *E*. *coli*, plasmid *pTET151-7475 TR* [29] was used as a template for all mutagenesis reactions.

**Table 2:** Plasmids and primers used in this study.

Plasmids	Relevant description1			Source or reference	
pMKK224	pSWU30 with P_*oar*_:*bacM*. Template for site-directed mutagenesis			[29]	
pDMZ106	pSWU30 with P_*oar*_:*bacM* with a stop codon at aa 131. For construction of EH106			This study	
pDMZ171	pSWU30 with P_*oar*_:*bacM* I124D/F125R. For construction of EH171			This study	
pDMZ175	pSWU30 with P_*oar*_:*bacM* L35E. For construction of EH175			This study	
pTET151–7475 TR	Expression plasmid for His-tagged BacM protein lacking the N-terminus. Template for site-directed mutagenesis.			[29]	
pTET151–7475 N-Hydro	Expression plasmid for His-tagged BacM protein lacking the N-terminus with an L35E mutation.			This study	
pTET151–7475 C-Hydro	Expression plasmid for His-tagged BacM protein lacking the N-terminus with I124D/F125R mutations.			This study	
pTET151–7475 Δ-Cterm	Expression plasmid for His-tagged BacM protein lacking the N-terminus with a stop codon at aa 131.			This study	
**Primers**			**Sequence**		
BacM L35E		CCACACGCTC**GA**GGGCAAGGGGAG			
GC—BacM L35E		CTCCCCTTGCCC**TC**GAGCGTGTGG			
BacM I124D/F125R		GACCGCGGTGTC**GA**C**CG**CGAGGGCTCGCTG			
GC—BacM I124D/F125R		CAGCGAGCCCTCG**CG**G**TC**GACACCGCGGTC			
BacM 131 stop		GGCTCGCTGAAG**TA**GGAGAACCTGGGC			
GC—BacM 131 stop		GCCCAGGTTCTCC**TA**CTTCAGCGAGCC			

^a^. **Bolded** residues are mismatches for site-directed mutagenesis.

### Protein Expression in *E*. *coli*

Plasmids containing either N-terminally truncated wild type *bacM* or *bacM* mutant sequences generated through site-directed mutagenesis were transformed into *E*. *coli* strain BL21 (DE3). Transformants were selected on LB agar containing ampicillin. To induce protein expression, overnight cultures were inoculated into fresh LB media and grown at 37°C to an OD_600nm_ ~0.6. 1 mM isopropyl β–D-1-thiogalactopyranoside was added to the cultures and the cells were cultivated at 16°C overnight. Cells were harvested and stored at -80°C. For protein purification, cells were thawed, re-suspended in lysis buffer (100 mM NaH_2_PO_4_, 10 mM Tris-HCl pH 8.0, 8 M urea) and incubated at RT for 1 h. Cell debris was removed by centrifugation (10 min 10,000 x g) and the BacM-containing supernatant was added to equilibrated Ni-NTA agarose beads (Invitrogen, Carlsbad, CA) and incubated for 1 h at RT. Beads were washed with wash buffer (100 mM NaH_2_PO_4_, 10 mM Tris-HCl pH 6.4, 8 M urea) and eluted with elution buffer (100 mM NaH_2_PO_4_, 10 mM Tris-HCl pH 5.8, 8 M urea). Purified protein aliquots were stored at 4°C.

### BacM Polymerization Assay

Electron microscopic visualization was used to assess the polymerization of wild type BacM and various mutants. Purified BacM in 8 M urea was diluted to ~10 μM (unless otherwise indicated) and dialyzed overnight against various buffers and solutions. For wild type BacM the following conditions were used: 20 mM Tris-HCl, pH 7.5 (control); 20 mM glycine-HCl, pH 3.5 and 4.5; 20 mM glycine-NaOH, pH 8.5, 9.5 and 10.5; 20 mM Na_2_HPO_4_-NaOH, pH 11.5; 20 mM NaH_2_PO_4_, pH 6.0 (pH adjusted with citric acid); 20 mM Tris-HCl, pH 7.5 containing 0.25, 0.5, 1 or 2 M NaCl; or 1, 2, 3 or 4 M urea. To test the specific effects of glycine, BacM was dialyzed against 20 mM Tris-HCl, pH 7.5, CHES, pH 9.5 or CAPS, pH 10.5 with or without 20 mM glycine (pH adjusted after the addition of glycine to final pH). For the re-constitution of fibers from various mutant BacM, protein solutions were diluted to 10 μM with 20 mM Tris-HCl, pH 7.5 and dialyzed against this buffer overnight.

### Circular Dichroism (CD) Spectroscopy

Wild type or BacM mutant proteins (as indicated in the text) were purified from *E*. *coli* and dialyzed against 20 mM Tris-HCl, pH 7.5 to a final concentration of 0.5–1 mg/ml. Under these conditions, wildtype BacM, but not mutant BacM, formed polymers. CD spectra were recorded from 320–180 nm in continuous mode at a scanning speed of 50 nm/min using a Jasco J-810 Spectropolarimeter and the Spectra Manager Software (Jasco Inc., Easton, MD). For comparison, the α-helix-only myosin A-tail interacting protein, MTIP was used [32,33]. Data points were subtracted from a background reading of the buffer, and the molar ellipticity was calculated. Data are presented as the molar ellipticity (deg cm^2^/dmol) at each wavelength (nm).

### Fourier Transform Infrared (FTIR) Spectroscopy

250 μg purified BacM fibers were centrifuged at 6°C in a tabletop centrifuge (10,000 x g for 10 min). After washing the pellet with water, the fibers were re-suspended in 100 μl de-ionized water and 80 μl of the sample was evaporated to dryness on the surface of a germanium crystal under a stream of dry nitrogen gas. A Bruker Vertex 70 FTIR spectrometer (Bruker, Billerica, MA) equipped with a TGS detector was used to collect in the mid-infrared region (4000 to 800 cm^-1^) 1024 scans in ATR mode at a resolution of 2 cm^-1^. To record the spectrum of the deuteriated BacM fibers, the sample was re-measured after a 30 min continuous exposure to a D_2_O-saturated nitrogen gas stream in a custom-made chamber containing the crystal. 16 scans each were used to record the HD exchange kinetics in 2 to 5 min intervals. To enhance absorption bands for analysis, Fourier self-deconvolution (FSD) of the OPUS software (version 6) was applied to spectra using a noise reduction parameter of 0.25 and a bandwidth of 18.7 cm^-1^. For comparison the antiparallel β-strand-containing porin Omp32 was used [34].

### *In silico* Modeling of BacM

Homology models of BacM were generated using the online servers QUARK [35], I-TASSER [36,37], Robetta [38], MUFOLD [39], MULTICOM [40,41], and Phyre2 [42]. All six servers were run using the default server settings. I-TASSER, MUFOLD, and MULTICOM each selected ten templates for threading of the target sequence, which was then divided into fragments. The fragments of the threaded sequence were then combined to build initial models, energy minimized, and clustered to generate five top models for each I-TASSER and MUFOLD prediction, and one top model for MULTICOM. The Robetta and Phyre2 servers generated top models based on traditional homology modeling, using a single template for each generated model. See S1 Fig. for the templates used by the five template-based servers.

The top I-TASSER model was selected for docking studies, based on its agreement with the repeat sequence prediction (see Results for rationale). The full-length model and a model truncated after Lys^130^, were docked using the ClusPro 2.0 server [43] and default settings with no restraints. The ClusPro server generated models were grouped into four categories based on the weighting of the interactions calculated: balanced, electrostatic-favored, hydrophobic-favored, and Van der Waals combined with electrostatics. Models were selected for their ability to form head-to-tail interactions that matched with *in vitro* microscopic observations. The changed solvent accessible surface areas for the various models were calculated using the Pisa server [44].

### Light and Fluorescence Microscopy

Light and fluorescence microscopy were performed essentially as previously described [29]. Briefly, for light microscopy, cells were grown in liquid culture to mid-logarithmic phase and spotted on a glass slide.Samples were imaged using a Nikon Eclipse 90i microscope with a 100x/NA 1.4 phase-contrast oil immersion objective (Nikon, Melville, NY). Individual images were recorded using an ORCA ER CCD camera (Hamamatsu, Bridgewater, NJ) and processed using the Volocity software package (PerkinElmer, Waltham, MA). For immunofluorescence microscopy, cells were grown in liquid culture and then placed in submerged culture and allowed to adhere overnight to autoclaved glass cover slips in 35 mm plastic dishes at 32°C. Cells were rinsed with phosphate magnesium (PM) buffer (20 mM Na-phosphate, 1 mM MgSO_4_, pH 7.4) and fixed for 40 min at room temperature with 4% paraformaldehyde diluted in PM buffer. Finally, cells were permeabilized with 0.2% triton X-100, lysozyme-treated (1 mg/ml), washed, blocked with 2% BSA, and probed with an affinity-purified anti-BacM antibody as described [29]. Samples were imaged as above, using a DAPI or TRITC filter cube.

### Electron Microscopy

Polymerized BacM was applied to glow-discharged carbon-coated 400 mesh copper grids (Electron Microscopy Sciences, Hatfield, PA) and negatively stained with un-buffered 2% uranyl acetate. The grids were examined in a Philips BioTwin CM120 microscope (FEI, Hillsboro, OR) or a Hitachi 7600 microscope (Hitachi High Technologies America Inc., Schaumburg, IL). Digital images were captured on 4k x 4k CCD cameras (Gatan, Warrendale, PA).

## Results

### Circular Dichroism and Infrared Spectroscopy of Purified BacM Fibers indicate a Primarily Parallel β-sheet Structure

**Fig 1:**
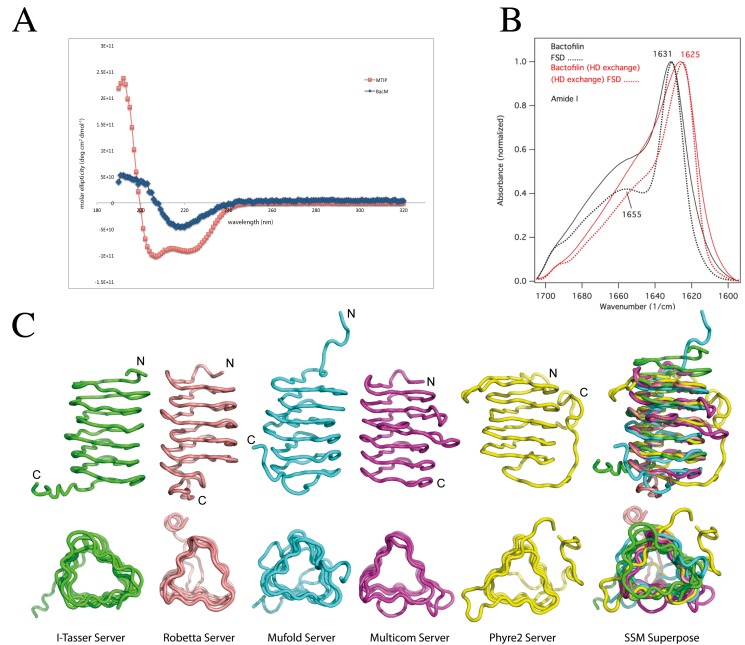
Generation of an *in silico* BacM model. (**A**) The CD spectrum of isolated purified BacM fibers indicates a β-structure-only protein devoid of α-helices. (**B**) The observed peak at 1631 cm^-1^ of the amide I region of the FTIR spectrum of purified BacM fibers is likewise characteristic of β-sheets. No indications of α-helices are found as the peak between 1650 and 1655 cm^-1^ in the Fourier self-deconvoluted (FSD) spectrum disappeared upon deuteration. This behavior is indicative for unstructured regions. The shift of the major β-structure peak from 1631.9 cm^-1^ to 1625 cm^-1^ upon deuteration is characteristic of an “extended β-sheet” which is the result of intermolecular β-structure-like interactions of the polymerized BacM. (**C**) Ribbon diagrams of various BacM models generated by five different molecular modeling servers. All models predict that nearly the entire protein is formed by a left-handed β-helix with the non-structured flexible C-terminus variously oriented with respect to the barrel-shaped molecule. No α-helices are present in the models of the protein.

To begin characterization of the structure of the newly discovered bactofilin family of proteins, we purified fibers of exogenously expressed BacM from *E*. *coli* and analyzed them by circular dichroism (CD). The recorded spectrum indicated a minimum at 218 nm that is characteristic for a β-sheet-only structure [45], showing no resemblance to the CD spectrum of the control α-helix-only protein MTIP of *P*. *falciparum* (Fig. 1A). To confirm this assessment, we examined the fibers using Fourier transform infrared spectroscopy (FTIR) (Fig. 1B). The amide I region of the recorded spectrum revealed a prominent peak at 1631 cm^-1^, which is characteristic of β-sheets (for assignments of peaks to secondary structure elements see [46,47]). In agreement with the CD results, no indication of α-helices was found as the peak between 1650 and 1655 cm^-1^ in the spectrum (characteristic of α-helices or unstructured loops; [46,47]) disappeared when the protein was deuterated, which is typical for unstructured regions, here presumably coinciding with the proline-containing C-terminus. Moreover, the FTIR-measurements were consistent with β-strands forming parallel β-sheets, since the peak at 1690–1695 cm^-1^ that is characteristic of anti-parallel β-sheets such as the core structure of porin Omp32 [34] was very weak. Finally, the significant shift of the major β-structure peak from 1631.9 to ≈1625 cm^-1^ upon deuteration is characteristic of a so-called "extended β-sheet". This structure is the result of intermolecular β-structure-like interactions of aggregating or systematically polymerizing protein molecules (Fig. 1B, [46–48]). In summary, the results of the CD- and IR-spectroscopy indicated that BacM: (*i*) lacks α-helices, (*ii*) contains a small amount of unstructured secondary structure, and (*iii*) mainly consists of parallel β-sheets that through the formation of fibers (*iv*) form extended β-sheet structures.

### Generation of a 3-dimensional *in silico* Molecular Model of Mature BacM

To further explore the structure of bactofilins, we used multiple homology modeling servers to build a model of BacM lacking the proteolytically removed N-terminal region. Most of these servers were chosen from the top 10 competitors in the latest CASP competition for template-based modeling [49]. We used six servers, which operate by three different modeling approaches: single-template-based modeling; multi-template, threading-based modeling; and *ab initio* modeling. Reference structures were chosen automatically by the modeling programs, primarily based on percent sequence identity with the target and other scoring criteria unique to each server. For single-template-based modeling the Phyre2 server [42] used the trimeric LpxA-like protein YdcK from *Salmonella cholera* as template [DOI:10.2210/pdb2f9c/pdf], while the Robetta server [38] used the human dynactin p27 subunit [PDB 3TV0] [50]. For multi-template, threading-based modeling, we used 3 servers: I-TASSER [36,37], MUFOLD [39], and MULTICOM [40,41]. These servers selected ten unique templates, threaded the query sequence into the structures, generated fragments, and reassembled the fragments to build models. A sixth server, QUARK, was used to generate an *ab initio* model of the entire BacM protein [35]. A comparison of the top models from the template-based modeling servers are presented in Fig. 1C and the sequences of the templates used by the various servers can be found in S1 Fig.

All template-based modeling servers produced top models with a type-T, left-handed β-helix (LBH) fold, consistent with the CD- and IR-spectroscopy data (Fig. 1A, B). In contrast, the QUARK-generated *ab initio* model (not shown) predicted a β-sandwich or β-roll structure containing unstructured regions. As this model disagreed with the spectroscopy data, it was not considered further. A superposition of the top models generated by each of the five, template-based servers is shown in Fig. 1C. All models were essentially in agreement regarding the overall structure of BacM, converging on related structures despite the variety of the templates and modeling methods used. Consistent with our experimental observations, all template-based servers predicted that the majority of the protein is made of β-sheets and lacks α-helices. This model of a β-stranded structure would thereby represent a structural motif that has not been previously demonstrated in any bacterial cytoskeleton protein. As expected from the sequence alignments, the highest degree of confidence for this structure prediction lies in the highly conserved bactofilin domain. No good template structure was identified for the short C-terminus due to the highly flexible nature predicted for this region, and the highest variability between the models from the different prediction programs was in this region (Fig. 1C). In summary, the template-based models indicated that BacM (*i*) lacks α-helices, (*ii*) contains a small amount of unstructured secondary structure, and (*iii*) mainly consists of parallel β-sheets that form a characteristic LBH fold structure.

### Sequence Analysis reveals a Repeated Motif Common to Bactofilins, which predicts the Stacking of Hydrophobic Residues

**Fig 2:**
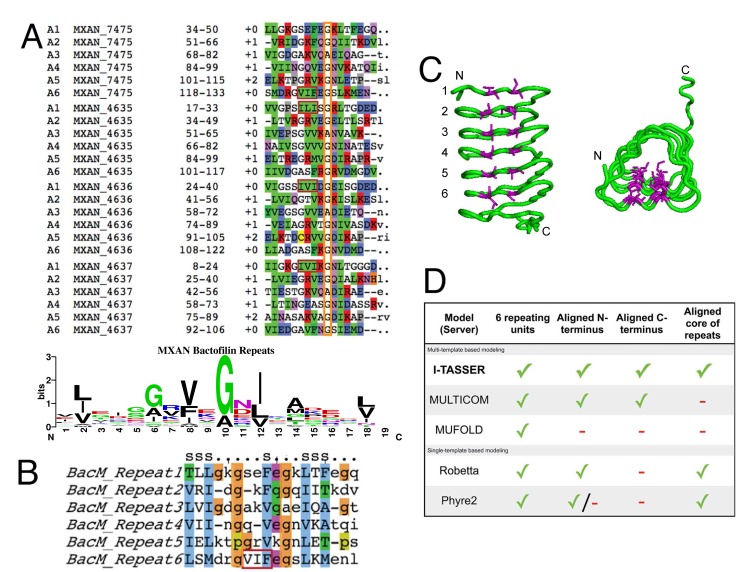
Bactofilins contain repeat regions with conserved hydrophobic residues within β-strands. (**A**) Alignment of the repeat regions in the 4 bactofilin paralogs of *M*. *xanthus* identified by HHrepID reveal that each paralog contains 6 repeats (top); Sequence logo showing conservation of residues at each position in the repeat, generated using the server at weblogo.berkeley.edu (bottom). (**B**) Repeat regions of BacM are aligned, revealing the highly conserved hydrophobic residues. S: β-strand; Orange box: highly conserved Gly residue; Red box: long second-strand anchor. (**C**) Conserved hydrophobic residues align along the β-sheet sides of the solenoid structure. Side and top views of the ribbon diagram of the I-TASSER BacM model with the first two hydrophobic residues of each repeat highlighted in purple. (**D**) Table of parameters used to judge the quality of the predicted models by the five template-based servers. Models were evaluated based on the number of repeats and their alignment as well as ordered N- and C-termini. Checks and minuses denote that a given model possesses or lacks a given quality, respectively.

While all template-based models for BacM are generally similar in that they predict a β-solenoid structure of six stacked repeat-elements, there are differences in the specific details regarding alignment and lengths of the β-strands. In order to determine if any of these models appear more likely to represent the structure of BacM than the others, we undertook a bioinformatics approach. To identify potential repeating units, we analyzed the sequences of the four bactofilin paralogs of *M*. *xanthus* using the HHrepID server [51] to identify conserved repeats. The server used BLAST to search for similar sequences to aid in identification of repeat sequences, and six repeats were identified for each bactofilin. The six repeats of each of the four bactofilins are aligned in Fig. 2A and A sequence logo, graphically representing sequence conservation at different positions in the repeat, was generated using the WebLogo server [52,53]. For clarity, we slightly adjusted the output so that the beginning of each repeat started with a β-strand and ended with a turn that would lead into the next strand (Fig. 2B). As seen in the alignments, the length of each repeating unit varies from 16 to 18 residues, and gaps in the alignment are all near the first conserved glycine of the repeat, suggesting variation in the length of the loop between strands (see below). We identified several highly conserved residues within these repeats, particularly hydrophobic residues which appear to anchor each β-strand to neighboring strands, and glycine residues that are located at the turns. From this sequence analysis and our spectroscopy data, we expect the structure to have repetitive units consisting of a β-strand anchored by two hydrophobic residues (Leu/Ile/Val), followed by a turn of varying length that contains a highly conserved Gly, followed by a shorter β-strand anchored by one hydrophobic residue (Val or Phe), followed by a shorter turn with a conserved Gly/Ala, and ending with a longer β-strand with two conserved hydrophobic residues (varying) and short turn. This sequence pattern is clearest and most highly conserved for repeat number 4 (Fig. 2B) and also conserved among bactofilins of multiple species, suggesting it is important to the function or structure of bactofilins (S2 Fig.). The repeat pattern identified by sequence alignment, conserved hydrophobic positions, and repeat length match well with the predicted β-solenoid structure output of the modeling servers (Fig. 1C).

Using our bioinformatics analysis, we next evaluated the models we had generated (Fig. 1C); we first evaluated the models for incorporation of 6 repeating units. All single-template and multi-template-based models contained this feature, supporting the strength of this prediction. We next determined whether the β-strands and conserved anchoring residues were aligned, as expected in the core of the model. We found that I-TASSER, Robetta, and Phyre aligned the β-strands consistent with the prediction from the sequence alignment, while the MULTICOM and MUFOLD models had frame-shifts of the repeats. In the MULTICOM model, the last two repeats were shifted by one turn, with the strand of a preceding repeat shifted into a loop. For the MUFOLD model, the shift occurred due to a loop insertion in a strand of repeat 4, shifting the alignment of subsequent repeats. Our final criteria were to evaluate the N- and C-terminal repeats and determine if they were aligned and if they stacked well, or were disordered. The quality of the N- and C-terminal repeats in terms of their order and alignment of the repeats is important because we expected that the monomers dimerize in a head-to-tail manner to form filaments and then laterally associate to form fibers (see below). If the N- and C-terminal repeats are not well ordered or properly aligned, then subsequent docking attempts would likely produce results inconsistent with our electron microscopic observations of BacM forming straight filaments. The N-termini of the I-TASSER, MULTICOM, and Robetta structures were correctly aligned and stacked. Additionally, the Phyre2 model had a well-ordered N-terminus, however, the second strand of the first repeat was shifted into the turns, and therefore was not correctly aligned. The first strand of the MUFOLD N-terminal repeat did not lay down on top of the second repeat and would interfere in docking. Looking at the C-terminus, many models were disordered, likely due to the C-terminal domain with no known homology to any structure. To determine which models to use in subsequent protein docking analysis, we evaluated how well the last repeat of the bactofilin core was ordered and aligned. In the Robetta model, the C-terminal repeat did not lie flat against the other repeats and the rest of the C-terminus was in a position that would block dimer formation. Both the Phyre2 and MUFOLD models had a poorly ordered C-terminal repeat. The MULTICOM and I-TASSER models both had ordered C-termini. In addition to a completely ordered sixth repeat, the I-TASSER model generated the first strand of what would be an unidentified seventh repeat. It is possible that this partial “pseudo repeat” exists, as there is a Leu and Thr that could fit into the sequence conservation and repeat alignment. However, our analysis using HHrepID did not identify these residues as part of a seventh repeat, and it is not currently possible to tell whether this additional strand is present or if it only is an artifact of the model building process. Despite this uncertainty, however, we chose to proceed with our experiments using the I-TASSER model, as this model was superior for accommodating stacking of the hydrophobic residues in the conserved repeat regions, and for having well-ordered N- and C-termini (Fig. 2C). A table comparing the various models in terms of the qualities discussed above is provided in Fig. 2D.

### BacM assembles *in vivo* and *in vitro* into Filaments, Fibers, and Ribbons

**Fig 3:**
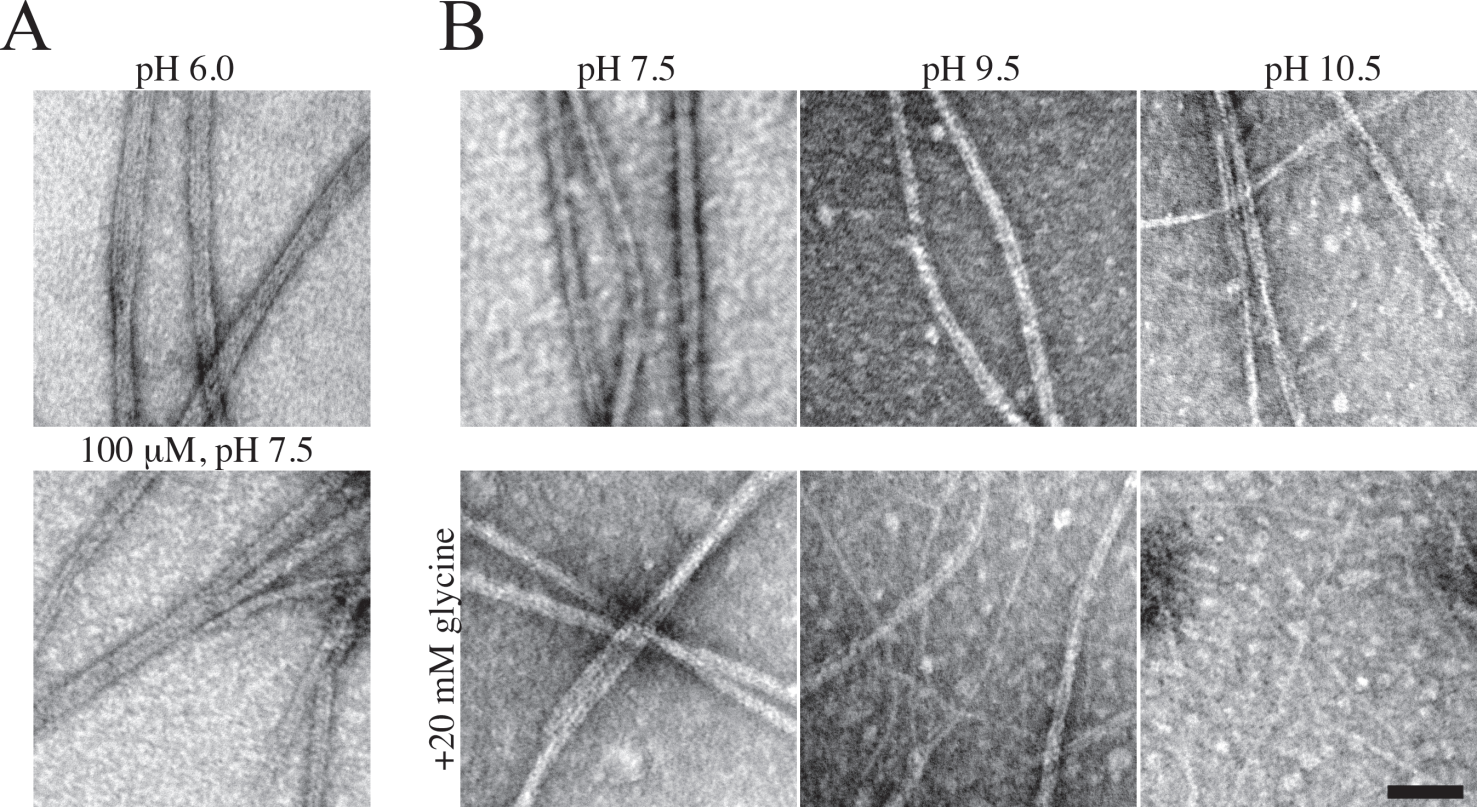
Structure of BacM fibers, ribbons, and filaments after dialysis against various buffers. Exogenously expressed and purified BacM polymerizes into different structures upon dialysis against different buffers. **(A)** In a phosphate-citric acid buffer (pH 6.0, top), or at high concentrations (100 μM, bottom), large ribbon-like structures were observed. (**B)** In Tris, CHES or CAPS buffers (pH 7.5, 9.5 or 10.5, respectively), 10 nm fibers resembling those isolated from *M*. *xanthus* cells predominate (top row). The addition of 20 mM glycine to the buffers (bottom row) favors the formation of 3 nm filaments at increasing abundance with increasing pH. Scale bar = 50 nm.

Previous electron microscopic examinations had revealed that BacM fibers isolated from cells were uniform, ~10 nm wide structures. Closer inspections of these fiber structures showed that at their ends they were occasionally frayed exposing thinner roughly 3 nm wide filaments [29]. Although these thinner filaments could not be isolated from cells *per se*, these observations suggested that *in vivo* the protein might polymerize into thin filaments, which associate into the 10 nm BacM fibers. To understand these processes better and to test whether exogenously overexpressed BacM could be used to recapitulate them *in vitro*, we purified the protein expressed in *E*. *coli* under denaturing conditions (8 M urea) and reconstituted BacM polymers by removal of the urea through dialysis (Fig. 3). When diluted to ~10 μM and dialyzed against Tris at pH 7.5, BacM was primarily observed as native-like fibers about 10 nm wide (Fig. 3B). Occasionally, thicker, multi-stranded ribbon structures were observed. These ribbons were more prevalent when BacM was dialyzed against a pH 6.0 buffer, or when higher protein concentrations (>100 μM) were used (Fig. 3A). Both fibers and ribbons appear to comprise multiple filaments, and we observed individual filaments of about 3 nm following dialysis against a strongly alkali glycine buffer (S3 Fig., pH 10.5). Under these buffer conditions, BacM assembles exclusively into the 3 nm filaments. When dialyzed against a less alkali buffer (pH 9.5), a mixture of 3 nm filaments and 10 nm fibers was observed, and 10 nm fibers were exclusively observed following dialysis against a pH 8.5 glycine buffer (S3 Fig.). While glycine is an appropriate buffer in this pH range [54], it is also a charged molecule. Thus, glycine may contribute to BacM filament formation by acting as a counterion, leading to a reduction in longitudinal binding. Such an effect was recently reported for the disassembly of microtubules in spermine-containing buffers [55]. To separate out the possible contributions of glycine as a buffer, and glycine as a counterion to BacM, we dialyzed BacM against Tris, pH 7.5, CHES, pH 9.5, and CAPS, pH 10.5 with or without glycine present. At all pH tested, 10 nm fibers were recovered in buffers lacking glycine (Fig. 3). The addition of glycine to each buffer favored recovery of 3 nm filaments, with exclusively filaments in the pH 10.5 buffer, a mixture in pH 9.5 buffer, and partial “unraveling” of fibers at pH 7.5, similar to the results of glycine alone, indicating that glycine is most likely exerting its effect as a counterion. To test if the 3 nm filaments constitute BacM polymers that form due to the presence of glycine, or comprise the elementary polymeric structure of BacM, we generated 10 nm fibers in a Tris, pH 7.5 buffer and subsequently dialyzed these fibers against an alkali, glycine containing buffer. These 10 nm fibers separated into 3 nm filaments (S4 Fig.). Together, these data suggest that BacM polymerizes into 3 nm filaments and that these filaments are either unstable or possess a high propensity to bundle into fibers, and that, under high protein concentrations, this interaction can lead to higher-ordered ribbon-like structures.

### The BacM Monomer Model predicts Hydrophobic Patches Important for Filament Formation and a Charged Surface

**Fig 4:**
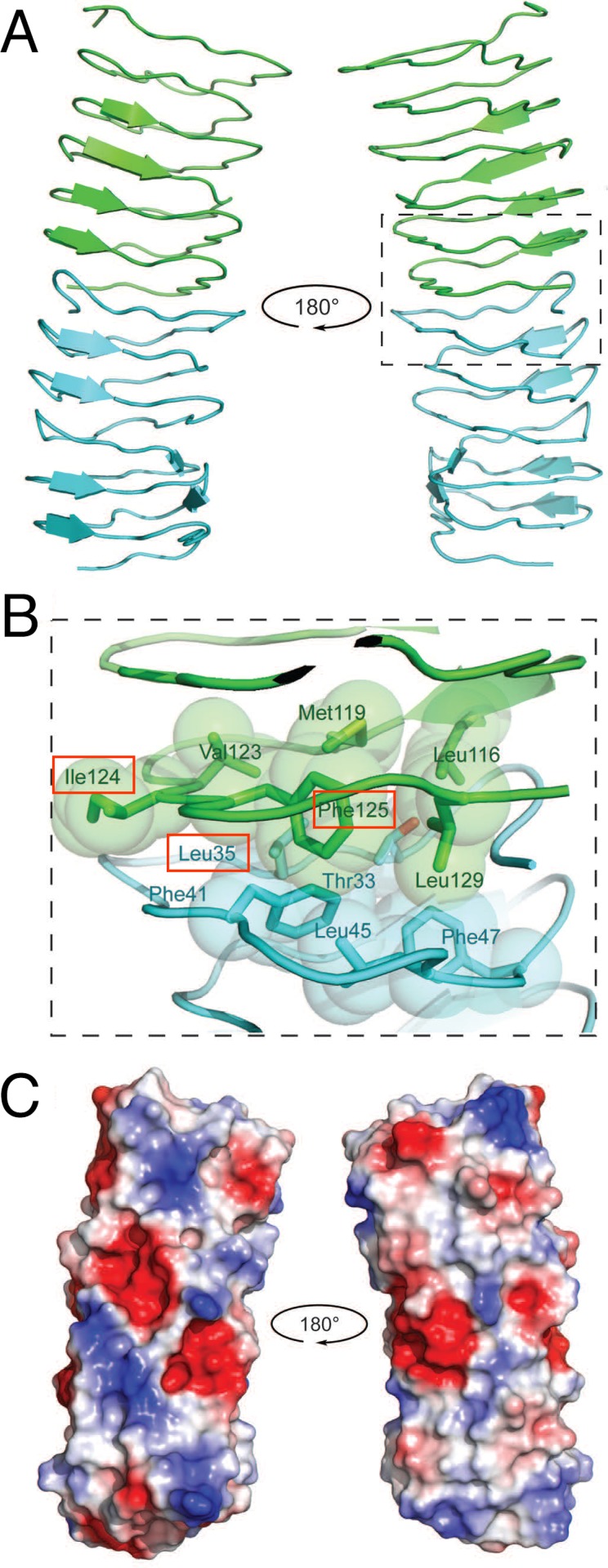
Modeling of the dimerization domain of bactofilin predicts hydrophobic interface and charged surfaces. (**A**) The bactofilin domain from the I-TASSER model was modeled using ClusPro 2.0 and predicts head-to-tail dimerization. Individual BacM monomers are represented as blue and green ribbon structures, and the dimer is presented as rotated 180^o^. (**B**) The interface between two bactofilin-domains is predicted to contain continuous stacking of hydrophobic subunits within β-strands, with highly conserved residues at key positions to form a hydrophobic pocket. The residues mutated in this study are highlighted with red boxes. (**C**) An electrostatic surface map of the dimer model reveals patches of charged residues that are solvent exposed. Negatively charged areas are shown in red, positively charged areas in blue, and areas that are charge neutral are shown in white.

Since BacM forms homo-monomeric filaments and fibers *in vivo* [29] and, like all bactofilins studied, similar fibers *in vitro* (see also [27]), we modeled the protein-protein interface responsible for dimerization using the ClusPro 2.0 docking server [43] in conjunction with our electron microscopic data (Fig. 4). The BacM model indicated a monomer size of roughly 3x3x3 nm and the electron microscopic data revealed ~3 nm wide filaments, suggesting an end-to-end stacking of monomers. Additionally, the extended β-sheet structure observed by FTIR suggest the most likely arrangement was a head-to-tail dimer. Using the best-predicted model from I-TASSER, we performed docking calculations to attempt to identify residues important to dimer formation.

**Fig 5:**
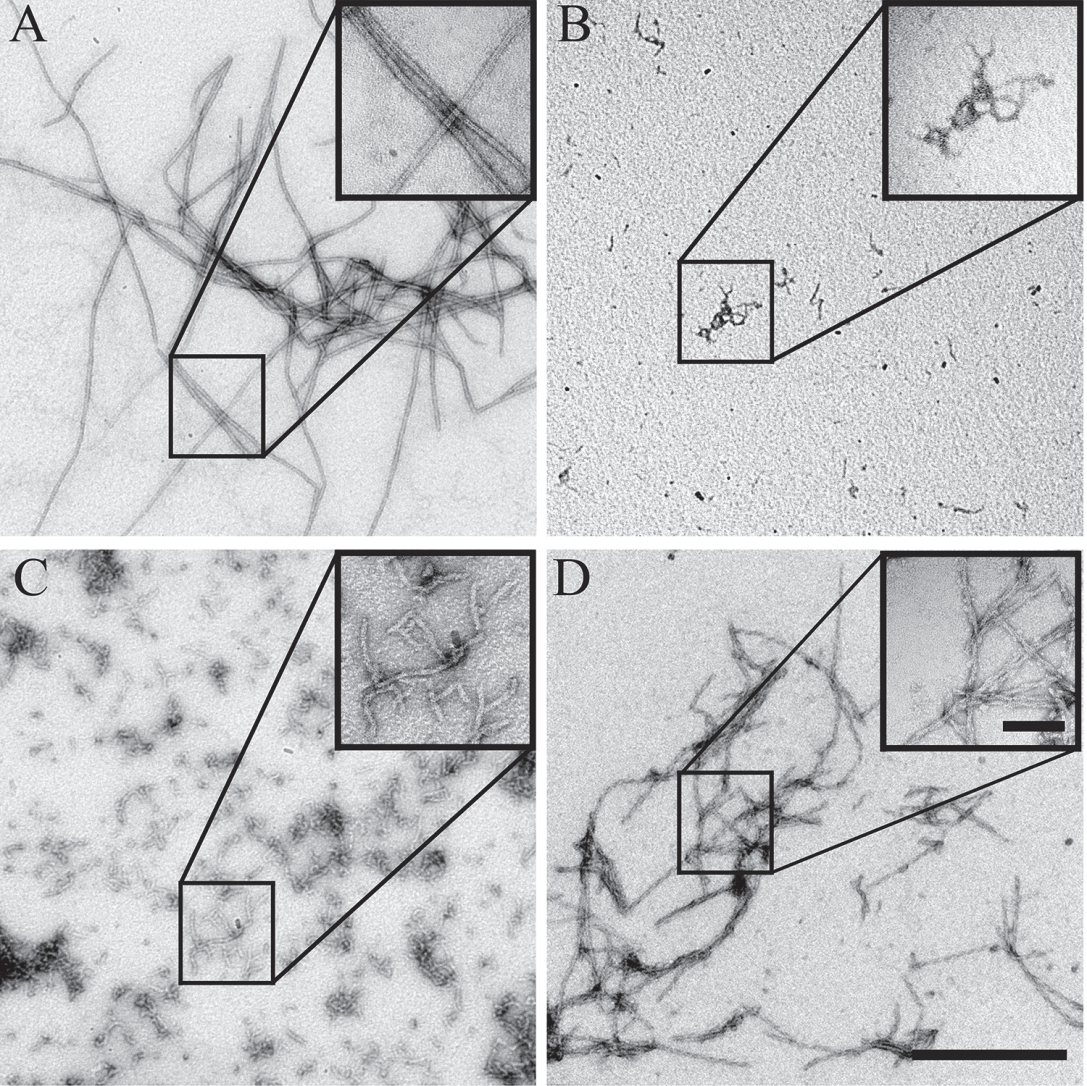
Evaluation of polymerization and fiber formation of recombinant mutant forms of BacM. While the wild type (**A**) and the C-terminal truncation mutant (**D**) are able to polymerize, the L35E (**B**) and the I124D/F125R (**C**) mutants are no longer able to form fibers and aggregate instead. (**D**) Despite their ability to polymerize, the C-terminal truncation forms fibers that show a distinct aberrant “braided” morphology when compared with the smooth wild type fibers. The scale bar in the large field is 0.5 μm, while the scale bar in the inset is 100 nm.

We proceeded to dock the full-length I-TASSER model, resulting in a head-to-tail dimer that involved the C-terminal residues Met^131^ to Ala^139^, which included the “pseudo-seventh repeat.” However, it is known that this C-terminal region of BacM is not necessary for filament formation. Fibers isolated from *M*. *xanthus* contain a high proportion of proteolyzed BacM [29], which lacks this “pseudo-repeat” region and the C-terminal residues identified by the ClusPro server to be at the interface; therefore, we reasoned that the bactofilin domain alone is sufficient for polymerization. To experimentally test this prediction, we expressed the bactofilin domain alone in *E*. *coli* and purified it under denaturing conditions. Upon dialysis into a polymerization buffer, we observed robust polymerization of the protein, though the architecture of the fiber was twisted and highly kinked, and filaments tended to be shorter relative to the wild type protein (Fig. 5D). This suggests that the C-terminus is indeed dispensable for polymerization but contributes to the stability of the fiber, and the lateral packing of filaments, which are responsible for the “smooth” appearance of the wild type fibers.

Based on the *in vivo* and *in vitro* data, which indicated that the C-terminus is not essential for polymerization, we used only the bactofilin domain for docking of the I-TASSER model. Using the ClusPro 2.0 server, we generated a best-balanced model that resulted in head-to-tail dimer formation (Fig. 4A). We found that the interface of the head-to-tail dimer is largely mediated by hydrophobic patches at the N- and C-termini of each monomer: Thr^33^, Leu^35^, Leu^45^, and Phe^47^ at the N-terminus, and Leu^116^, Met^119^, Val^123^, Phe^125^, and Leu^129^ at the C-terminus (Fig. 4B). The changed solvent accessible surface area (SASA) was ~1307 Å. Additionally, the top head-to-tail model was arranged so that the repeats continued to stack in order with strand 1, repeat 1 of monomer 1 found on the same side of the solenoid as strand 1, repeat 1 of monomer 2, allowing a continued stacking of repeat units, mediated by the conserved hydrophobic residues, from one monomer to the next, throughout the filament.

We next investigated the surfaces of the BacM dimer to predict how they could facilitate lateral interactions. As can be seen from the electrostatic surface maps (Fig. 4C), the exposed surface of BacM has strips of positively and negatively charged residues that could interact with other filaments to form higher ordered fibers. These oppositely charged surface areas could interact laterally, explaining the formation of the 10 nm wide fibers and why the thinner filaments are virtually never found. Additionally, when attempting to model a head-to-tail dimer interface using the ClusPro server, a result of two monomers interacting laterally *via* these electrostatic patches was a common result (not shown).

In summary, these modeling data predict that the bactofilin domain of BacM is sufficient for polymerization, and polymerizes into filaments in a head-to-tail fashion mediated by patches of hydrophobic residues at the N- and C-termini. These filaments are likely further stabilized by lateral electrostatic interactions that result in the formation of fibers, creating highly stable structures.

### Wild Type BacM forms Stable Filaments *in vitro* and Filament Formation depends on the Hydrophobic Patches of the Predicted Interface Domain

Sequences containing hydrophobic residues at specific locations on the solenoid are predicted to mediate interactions between repeat subunits within one BacM monomer. In order to test the prediction of our dimer model that these hydrophobic repeats also mediate interactions *between* subunits, we performed an *in vitro* assembly assay. We expressed His-tagged constructs encoding the bactofilin domain and C-terminus of BacM in *E*. *coli*, and purified the protein under denaturing conditions (8 M urea). We expressed wild type BacM and two BacM mutants with hydrophobic residues (within repeat 1 and repeat 6 (Fig. 2B)) mutated to charged residues (L35E and I124D/F125R). These residues were selected for mutation due to their predicted location at the dimer interface, and for their high degree of conservation. These proteins were purified under denaturing conditions (8 M urea). To ensure that these mutants were properly folded, urea was removed by dialysis, and the proteins were analyzed by CD spectroscopy. The recorded spectra closely matched that of wild type BacM, indicating a β-strand-only structure (S5 Fig.). To assay for polymerization, the mutants were diluted to ~10 μM, and urea was removed by dialysis. Spontaneous fiber formation of the dialysate was monitored by negative staining (Fig. 5). Wild type BacM formed relatively uniform, ~10 nm fibers, while filament formation was completely abolished for both mutants. Small aggregates of ~3 nm wide particles in preparations of both mutants were observed. This observation is consistent with BacM subunits that fail to polymerize into filaments, but are still able to laterally associate, or alternatively, the formation of short filaments (Fig. 5B and C). To discriminate between these two hypotheses, these mutants were dialyzed against an alkali glycine-containing buffer to reduce lateral interactions (Fig. 3). In this buffer, these small protein aggregates were completely absent, consistent with an interpretation that the aggregates observed are monomers of BacM that are associating *via* lateral electrostatic charges, which can be disrupted by addition of glycine in an alkali solution (S6 Fig.).

**Fig 6:**
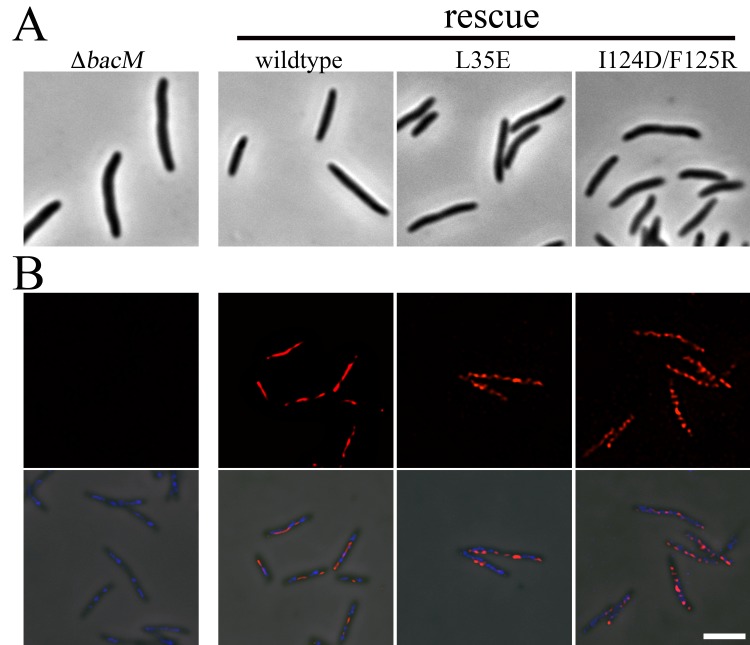
Mutations to putative BacM interface fail to rescue the morphological phenotype of a deletion mutant. (**A**) Images of Δ*bacM* cells or the indicated rescue strains were taken by phase contrast light microscopy. (**B**) The same cell lines were fixed and imaged by immunofluorescence microscopy with an anti-BacM antibody (red) and stained with DAPI (blue). Top panel, anti-BacM. Bottom panel, merge with phase contrast. Scale bar = 5 μm.

The Δ*bacM* mutant *M*. *xanthus* was described as having a morphological defect, where the typical rod-shape of the bacterium was crooked or kinked [29]. In order to test if the hydrophobic interface is required for proper BacM function *in vivo*, the L35E and the I124D/F125R mutations were introduced separately into a Δ*bacM* mutant of *M*. *xanthus* to test for rescue of the wildtype morhpology (Fig. 6). While introduction of wild type *bacM* restored the rod-shaped phenotype, both polymerization-defective mutants failed to rescue, although they were robustly expressed, as measured by immunoblot (Figs. 6A and S7A). When examined by immunofluorescence with an anti-BacM antibody, the overexpressed wild type *bacM* displayed the expected pattern of extended fibers across the length of the cell (Fig. 6B and [29]). BacM containing mutations to either interface domain appeared to form fiber-like staining to varying degrees. Unlike the wild type BacM, however, this staining appeared discontinuous across the cell. This effect was more severe with the I124D/F125R mutant than the L35E mutant, though they both failed to rescue (Fig. 6). BacM lacking the C-terminus was unable to be visualized in *M*. *xanthus*, as steady-state levels of the mutant protein were undetectable by immunoblot, possibly due to instability of the protein (S7B Fig.). Together, these data are consistent with our hypothesis that the bactofilin domain alone mediates polymerization of BacM into filaments, and that a hydrophobic interface is required for this interaction.

### Wild Type BacM forms Higher-ordered Fibers *in vitro* and Fiber Formation depends on Charged Surface Areas

**Fig 7:**
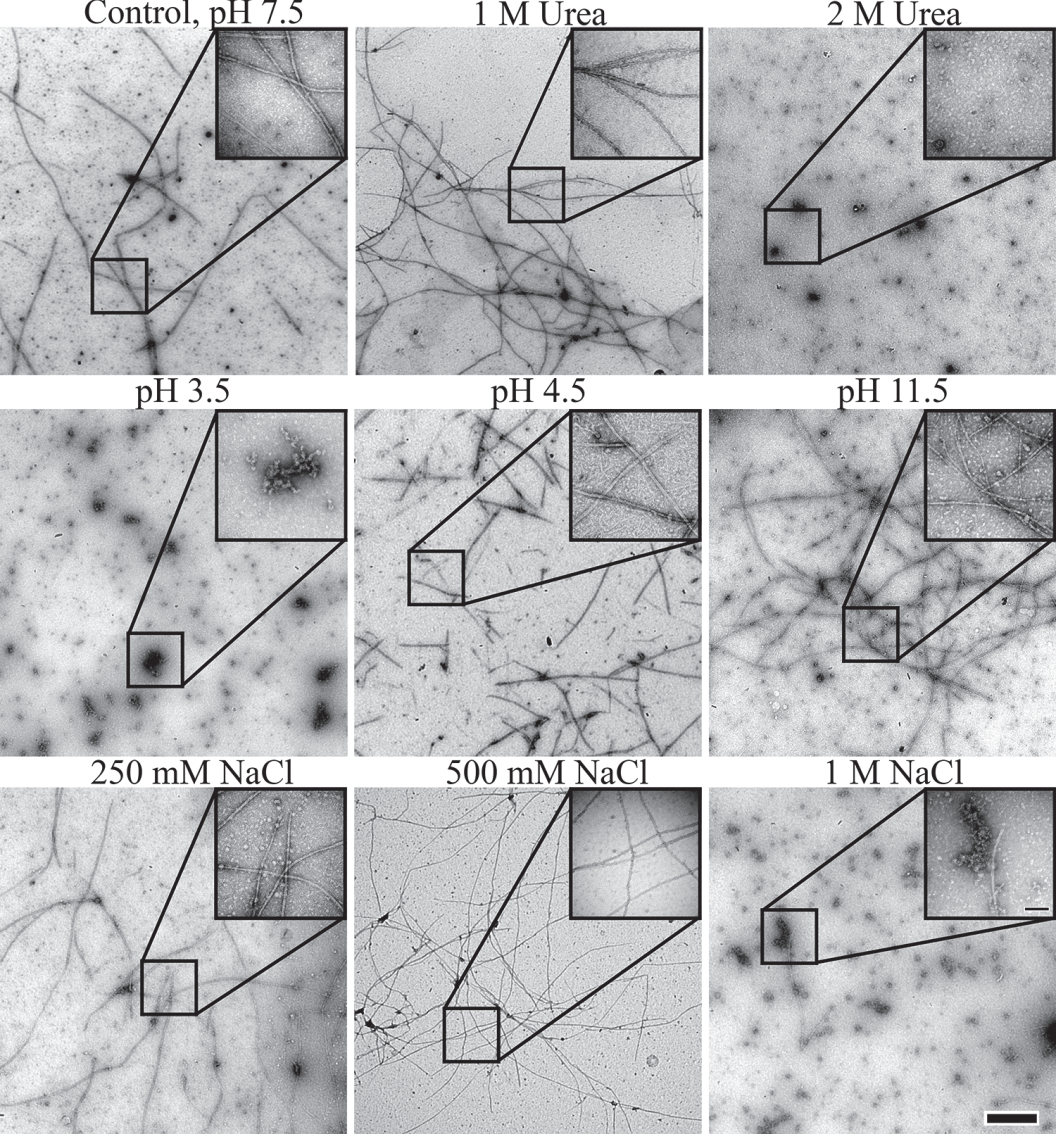
Evaluation of polymerization and fiber formation of recombinant wild type BacM in the electron microscope after various treatments. Fiber formation is robust and occurs under a wide variety of conditions. In the presence of chaotropic agents, such as urea, the wild type protein polymerizes at concentrations of up to 1 M (upper row), while polymerization is more sensitive to low than to high pH values (middle row) and does not occur at NaCl concentrations of 0.5 M and higher (lower row) indicating the importance of charge for the lateral association of BacM filaments. The scale bar in the large field is 0.5 μm, while the scale bar in the inset is 100 nm.

Although the 3 nm wide filaments appear to be the elementary polymerization unit of BacM (Fig. 3), and can be observed in alkali buffer containing glycine, they are seldom observed from BacM recovered from *M*. *xanthus* or *via* exogenous expression in *E*. *coli* in non-ionic buffers. Instead BacM is usually recovered as 10 nm wide fibers *in vivo* [29] and after over-expression and purification *in vitro* [27], indicating a high propensity for lateral association and fiber formation. In fact, the propensity for spontaneous polymerization and fiber formation is so robust that the isolation of exogenously expressed monomers from *E*. *coli* is only possible in the presence of high concentrations of urea (see Materials and Methods). In order to understand the fiber formation process better, we tested various conditions and their influence on fiber formation. For this, recombinant native BacM protein was purified under denaturing conditions (8 M urea) and then dialyzed against buffers with various salt concentrations, chaotropic agents or pH. The ability to form fibers under these conditions was assessed by negative staining and examination under the transmission electron microscope (Fig. 7). Under these conditions, spontaneous formation of native-like BacM fibers was observed when the urea concentration was reduced to 1 M or lower. In the absence of urea, BacM formed fibers across a wide range of pH in Tris buffer, and was only inhibited at extremes of pH (below pH 4.5 or above pH 11.5). Like urea, salt prevented fiber formation and polymerization at concentrations higher than 0.5 M. Unlike the samples dialyzed against glycine, however, 10 nm fibers were still observed, suggesting that NaCl does not similarly act as a counterion, preventing lateral interactions between filaments. Together these data showed that the β-sheet-based fold of BacM is extraordinarily stable and that lateral association for fiber formation is a robust process that occurs in a wide range of pH and even in the presence of chaotropic agents.

## Discussion

The fact that bacteria contain multiple cytoskeletal systems was realized only recently, and their discovery and rapid characterization owes much to macromolecular structural studies, as there is often low sequence homology to their eukaryotic counterparts. BacM is a newly discovered, uniquely bacterial, cytoskeleton protein [27] that forms highly stable, helically arranged polymers in *M*. *xanthus* [29]. Unlike bacterial cytoskeleton proteins of the MreB and FtsZ families [17,56], BacM polymerizes independently of nucleotides or cofactors. While this is similar to the bacterial IF homologous CreS, which also assembles in the absence of nucleotides or cofactors, CreS fibers disassemble when the pH is above 8.4, a hallmark of IFs [18]. Additionally, assembly of CreS, as other IF proteins, is mediated by its extensive coiled-coil domains. The resilience of BacM fibers at extreme pH, as well as the lack of predicted coiled-coils (or experimental evidence of α-helices at all), strengthens the notion that bactofilins comprise a novel class of bacterial cytoskeleton.

The propensity of BacM to spontaneously form highly stable fibers presents a great difficulty in crystallizing BacM protein for direct structural studies. As an alternative to X-ray crystallography, we computed a 3-D model of BacM in an attempt to predict important structural and functional residues and domains within the protein. The proposed model adopts a type-T left-handed β-helix fold (LBH), a highly regular and symmetrical structure with little variation in shape and size over the length of the domain [57], and is consistent with our experimental observations. So far all LBH X-ray structures that have been solved belong to bacterial proteins, a vast majority of which have enzymatic, particularly transferase, activities, composing the trimeric LpxA-like superfamily [58–60]. All but one LBH-containing proteins form homo-trimers (trimeric LpxA-like proteins), the only exception being the anti-freeze protein from the spruce budworm, which appears to be monomeric under physiological conditions [61]. Importantly, the LBH domain facilitates the trimerization of these proteins by laterally aligning into a bundle in the center of the structures. Although additional alignments resulting in LBH domain-mediated fiber formation is thought possible [62], it has so far not been reported for any protein under physiological conditions. The main reason being that the self-association of the LBH domain is likely restricted to trimerization by additional structural elements at the N- or C-termini of the proteins [63]. Consequently, it has been predicted that removal of these structural elements would expose the β-strands, leading to un-restricted self-assembly [62]. Analysis of the sequence of the N-terminally cleaved mature BacM shows that ~80% of the protein is formed by the LBH fold-containing DUF583 domain. Only 5 and 23 aa at the N- and C-terminus, respectively, are not part of this structure, likely limiting their influence to restrict polymerization. Since BacM forms 3 nm wide filaments *in vivo* and *in vitro*, the individual protein monomers are therefore most likely stacked head-to-tail within these filaments. This interpretation is in line with (*i*) the electron microscopic observations, (*ii*) our *in silico* models of the dimerization and filament formation, and (*iii*) the spectroscopic data that show the presence of extended β-sheet structures, which indicate that the β-sheets within the BacM polymer are highly oriented. Since these predicted 3 nm filaments are rarely observed *in vitro* and not recovered *in vivo*, it is clear that they either immediately interact with each other by forming the ~10 nm thick fibers or that filament polymerization and fiber bundling are coupled processes. This finding is reminiscent of eukaryotic microtubules, which are assembled by end-to-end interactions of dimer subunits to form protofilaments, which form a tube *via* longitudinal interactions between the protofilaments [64]. Analogous to our finding that lateral interactions of BacM filaments were disrupted by the presence of anionic glycine, it was recently reported that incubation of assembled microtubules with the cation spermine triggered the disassembly of microtubules into protofilaments, presumably by acting as a counterion, weakening the lateral interactions between protofilaments and favoring disassembly [55].

We have recently reported that the bactofilin domain is necessary [29] and show here that this domain alone is sufficient for fiber formation of BacM. Based on these findings, we computed models of the protein-protein interaction interface consistent with this earlier observation, as the interface region is entirely contained within the conserved bactofilin domain. Interactions between terminal hydrophobic regions are a novel structural principle for how proteins containing LBH folds can oligomerize. The hydrophobic nature of the BacM self-assembly is experimentally supported by *in vitro* examinations of BacM mutant proteins in which the identified critical amino acid residues of the interface domain have been replaced. These BacM mutant proteins are deficient for filament and fiber formation *in vitro* and are unable to rescue the morphological defect of a *M*. *xanthus* Δ*bacM* deletion strain. Moreover, these mutant strains also failed to display the fiber-like staining pattern *in vivo* when examined by immunofluorescence light microscopy. Light microscopic observation of these fiber-forming mutants showed that they failed to rescue the morphological phenotype of a *bacM* mutant indicating that the altered fiber morphology interferes with the biological function of the cytoskeleton protein.

The spontaneous polymerization of BacM under a wide range of conditions poses a fundamental problem for the bacterial cell. Namely, how the cells control this process. In earlier work, we had established that BacM is post-translationally cleaved, and proposed that this process is important for the control of polymerization [29]. Here we propose that the concentration of the protein is also a factor. During our *in vitro* polymerization experiments, we observed thick, multi-stranded ribbons of BacM that predominate at high protein concentration (~100 μM). These multi-stranded ribbons may explain the rod-like structures observed by immunofluorescence examination of BacM in ~20% of wild type cells [29]. Intriguingly, these thicker BacM rods are observed in virtually all cells upon overexpression of *bacM* [29], suggesting that at higher concentration, excess BacM filaments continue to bundle with existing fibers eventually forming thicker ribbons.

While the monomer model has high degree of confidence for the structure prediction of the highly conserved bactofilin domain, the structure and arrangement of the C-terminus is currently less clear. This domain most likely also accounts for the small portion of unstructured region measured in the FTIR experiments. This interpretation is in line with the observation that the C-terminus of BacM contains a cluster of five proline residues [29] that are usually indicative of unstructured regions of proteins [65,66]. Although these poly-proline-containing regions are “unstructured,” they have been identified as important elements engaging in the recruitment, recognition, and binding of other peptides and proteins [67]. Since it has been suggested that bactofilins act as scaffolds, exerting their morphogenic effect through the binding of PG-remodeling enzymes [27,28], this region of the protein could be an important binding site for these enzymes. Alternatively, the highly charged surface of BacM that contains negatively and positively charged patches could interact with such enzymes and other BacM-binding proteins through electrostatic interactions similar to the lateral BacM-BacM interactions described here. Finally, the C-terminus appears also to play a role in lateral interaction between individual fibers, because a truncated version of the protein polymerizes but forms characteristic kinked bundles that look distinctly different from the smoother bundles formed by the wild type protein.

The results presented in this study are the first prediction of a molecular model of the highly conserved bactofilin domain, the core structure of bactofilins, a novel family of bacterial cytoskeleton proteins [13]. Since fiber-forming proteins are intrinsically refractory to crystallization, so far only relatively few such proteins have been resolved at atomic resolution using X-ray crystallography, and in some cases, like actin, more unconventional approaches such as cryo-electron microscopy in conjunction with image analysis [68]. Using an *in silico* approach, we predict that the bactofilin domain has a LBH fold and polymerizes through the interaction of mainly hydrophobic residues into 3 nm thick head-to-tail monomer-containing filaments, while electrostatic interactions allow the protein to laterally bundle into 10 nm wide fibers. Both polymerization processes are extraordinarily robust and occur under a wide variety of conditions including high concentrations of urea and extreme pH. While *in silico* models are inherently speculative, our model has allowed us for the first time to identify important residues that are crucial for polymerization and fiber formation, thereby defining an unreported interaction interface for LBH-fold-containing proteins that may also be relevant for our understanding of potential amyloid-forming LBH-containing proteins commonly associated with severe human diseases [69–72]. Future mutational studies in conjunction with *in vitro* and *in vivo* examination of the polymerization behavior of BacM should further help to understand the structural principles guiding polymerization and fiber formation and eventually result in studies that will establish the atomic structure of this important novel class of bacterial cytoskeleton proteins.

## Supporting Information

S1 Fig Sequence-based alignment of templates used for homology modeling. The 30 structures used as templates to generate homology models by five servers were aligned using ClustalW. The sequences have been aligned with the BacM sequence residues 28–150. ClustalX coloring was applied to sequences in Jalview to denote amino acid conservation [73].
(PDF)

S2 Fig Bactofilin repeats are conserved between bactofilins of multiple species. 4 *M*. *xanthus* bactofilin paralogs are aligned with BacA and BacB of *C*. *crescentus* (CC_1873 and CC_3022, respectively), CcmA from *P*. *mirabilis* (PMI1961) and CcmA from *H*. *pylori* (HPG27_1480).
(TIF)

S3 Fig Structure of BacM fibers, ribbons, and filaments after dialysis in glycine buffers at different pH. Exogenously expressed and purified BacM polymerizes into different structures upon dialysis against 20 mM glycine buffer at various pH. At pH 8.5 10 nm fibers resembling those isolated from *M*. *xanthus* cells predominate (pH 8.5). Upon further increase of the pH, the 10 nm fibers are more and more replaced by 3 nm filaments (pH 9.5), which at pH 10.5 are the only observed form of polymerized BacM (pH 10.5). Scale bar, 50 nm.
(TIF)

S4 Fig Reconstituted 10 nm BacM fibers can be laterally separated by a pH change into 3 nm filaments. BacM was purified in 8M urea, and subsequently dialyzed against 20 mM glycine, pH 8.5. A sample was removed, applied to a copper grid, negative stained, and imaged by transmission electron microscope (left). The remainder of the dialysate was then dialyzed against 20 mM glycine, pH 10.5 and imaged as above (right). Scale bar = 25 nm.
(TIF)

S5 Fig Non-polymerizing mutants and C-terminal truncation mutant of BacM are properly folded. The indicated BacM mutants were purified as described in Materials and Methods, and dialyzed against 20 mM Tris, pH 7.5 to a final concentration of 0.5–1.0 mg/ml. These dialysates were examined by circular dichroism spectroscopy, as described in Materials and Methods. The α–helix-only protein MTIP was used as a control [32,33].
(TIF)

S6 Fig Polymerization-defective mutants of BacM form aggregates *via* lateral interactions. The indicated mutants of BacM were expressed in *E*. *coli* and purified in 8 M urea, and subsequently dialyzed against 20 mM Tris, pH 7.5 (left) or 20 mM glycine, pH 10.5 (right). Samples were applied to a copper grid, negative stained, and imaged by transmission electron microscope. While aggregates were ubiquitously found at pH 7.5, they were absent in samples prepared from the glycine buffer. Scale bar = 25 nm.
(TIF)

S7 Fig BacM L35E and I124D/F125R mutants have normal steady-state levels and the ΔC-terminus mutant fails to express. Lysates from the indicated strains of *M*. *xanthus* were separated by SDS-PAGE and immunoblotted with an affinity purified anti-BacM antibody [29]. **(A)** Lane 1: wildtype (DK1622); Lane 2: Δ*bacM* (EH301); Lane 3 Δ*bacM* with wildtype *bacM* rescue (EH344); Lane 4: Δ*bacM* with I124D/F125R *bacM* rescue (EH171); Lane 5: Δ*bacM* with L35E *bacM* rescue (EH175). **(B)** Lane 1: wildtype (DK1622); Lane 2: Δ*bacM* (EH301); Lane 3 Δ*bacM* with *bacM-*Δ*C-term* rescue (EH106). Unlabeled lanes are from strains not discussed in this report.
(TIF)

## References

[1] Shih YL, Rothfield L. The bacterial cytoskeleton. Microbiol Mol Biol Rev. 2006;70: 729–754. 16959967 10.1128/MMBR.00017-06 PMC1594594

[2] Gitai Z. Diversification and specialization of the bacterial cytoskeleton. Curr Opin Cell Biol. 2007;19: 5–12. 17178455 10.1016/j.ceb.2006.12.010

[3] Thanbichler M, Shapiro L. Getting organized—how bacterial cells move proteins and DNA. Nat Rev Microbiol. 2008;6: 28–40. 18059290 10.1038/nrmicro1795

[4] Graumann PL. Dynamics of bacterial cytoskeletal elements. Cell Motil Cytoskeleton. 2009;66: 909–914. 10.1002/cm.20381 19466751

[5] Löwe J, Amos LA. Evolution of cytomotive filaments: the cytoskeleton from prokaryotes to eukaryotes. Int J Biochem Cell Biol. 2009;41: 323–329. 10.1016/j.biocel.2008.08.010 18768164

[6] Cabeen MT, Jacobs-Wagner C. The bacterial cytoskeleton. Annu Rev Genet. 2010;44: 365–392. 10.1146/annurev-genet-102108-134845 21047262

[7] Erickson HP, Anderson DE, Osawa M. FtsZ in bacterial cytokinesis: cytoskeleton and force generator all in one. Microbiol Mol Biol Rev. 2010;74: 504–528. 10.1128/MMBR.00021-10 21119015 PMC3008173

[8] Celler K, Koning RI, Koster AJ, van Wezel GP. Multidimensional view of the bacterial cytoskeleton. J Bacteriol. 2013;195: 1627–1636. 10.1128/JB.02194-12 23417493 PMC3624557

[9] Popp D, Robinson RC. Many ways to built an actin filament. Mol Microbiol. 2011;80: 300–308. 10.1111/j.1365-2958.2011.07599.x 21362063

[10] Teixidó-Travesa N, Roig J, Lüders J. The where, when and how of microtubule nucleation—one ring to rule them all. J Cell Sci. 2012;125: 4445–4456. 10.1242/jcs.106971 23132930

[11] Goldman RD, Cleland MM, Murthy SN, Mahammad S, Kuczmarski ER. Inroads into the structure and function of intermediate filament networks. J Struct Biol. 2012;177: 14–23. 10.1016/j.jsb.2011.11.017 22120848 PMC3269975

[12] Ingerson-Mahar M, Gitai Z. A growing family: the expanding universe of the bacterial cytoskeleton. FEMS Microbiol Rev. 2012;36: 256–266. 10.1111/j.1574-6976.2011.00316.x 22092065 PMC4114309

[13] Lin L, Thanbichler M. Nucleotide-independent cytoskeletal scaffolds in bacteria. Cytoskeleton. 2013;70: 409–423. 10.1002/cm.21126 23852773

[14] Nogales E, Wolf SG, Downing KH. Structure of the alpha beta tubulin dimer by electron crystallography. Nature. 1998;391: 199–203. 9428769 10.1038/34465

[15] Bork P, Sander C, Valencia A. An ATPase domain common to prokaryotic cell cycle proteins, sugar kinases, actin, and hsp70 heat shock proteins. Proc Natl Acad Sci USA. 1992;89: 7290–7294. 1323828 10.1073/pnas.89.16.7290 PMC49695

[16] Jones LJ, Carballido-Lopez R, Errington J. Control of cell shape in bacteria: helical, actin-like filaments in *Bacillus subtilis* . Cell. 2001;104: 913–922. 11290328 10.1016/s0092-8674(01)00287-2

[17] Van den Ent F, Amos LA, Löwe J. Prokaryotic origin of the actin cytoskeleton. Nature. 2001;413: 39–44. 11544518 10.1038/35092500

[18] Ausmees N, Kuhn JR, Jacobs-Wagner C. The bacterial cytoskeleton: an intermediate filament-like function in cell shape. Cell. 2003;115: 705–713. 14675535 10.1016/s0092-8674(03)00935-8

[19] Bagchi S, Tomenius H, Belova LM, Ausmees N. Intermediate filament-like proteins in bacteria and a cytoskeletal function in *Streptomyces* . Mol Microbiol. 2008;70: 1037–1050. 10.1111/j.1365-2958.2008.06473.x 18976278 PMC2680258

[20] Waidner B, Specht M, Dempwolff F, Haeberer K, Schaetzle S, Speth V, et al A novel system of cytoskeletal elements in the human pathogen *Helicobacter pylori* . PLoS Pathog. 2009;5: e1000669 10.1371/journal.ppat.1000669 19936218 PMC2776988

[21] Fiuza M, Letek M, Leiba J, Villadangos AF, Vaquera J, Zanella-Cleon I, et al Phosphorylation of a novel cytoskeletal protein (RsmP) regulates rod-shaped morphology in *Corynebacterium glutamicum* . J Biol Chem. 2010;285: 29387–29397. 10.1074/jbc.M110.154427 20622015 PMC2937971

[22] Fenton AK, Hobley L, Butan C, Subramaniam S, Sockett RE. A coiled-coil repeat protein CcrP in *Bdellovibrio bacteriovorus* prevents cellular indentation, but is non-essential for vibroid cell morphology. FEMS Microbiol Lett. 2010;313: 89–95. 10.1111/j.1574-6968.2010.02125.x 20977494 PMC4803027

[23] Specht M, Schätzle S, Graumann PL, Waidner B. *Helicobacter pylori* possesses four coiled-coil-rich proteins that form extended filamentous structures and control cell shape and motility. J Bacteriol. 2011;193: 4523–4530. 10.1128/JB.00231-11 21642462 PMC3165534

[24] Gerdes K, Howard M, Szardenings F. Pushing and pulling in prokaryotic DNA segregation. Cell. 2010;141: 927–942. 10.1016/j.cell.2010.05.033 20550930

[25] Marchler-Bauer A, Anderson JB, Chitsaz F. Derbyshire MK, DeWeese-Scott C, Fong JH, et al CDD: specific functional annotation with the Conserved Domain Database. Nucleic Acids Res. 2009;37: D205–210. 10.1093/nar/gkn845 18984618 PMC2686570

[26] Hay NA, Tipper DJ, Gygi D, Hughes C. A novel membrane protein influencing cell shape and multicellular swarming of *Proteus mirabilis* . J Bacteriol. 1999;181: 2008–2016. 10094676 10.1128/jb.181.7.2008-2016.1999 PMC93611

[27] Kühn J, Briegel A, Mörschel E, Kahnt J, Leser K, Wick S, et al Bactofilins, a ubiquitous class of cytoskeletal proteins mediating polar localization of a cell wall synthase in *Caulobacter crescentus* . EMBO J. 2010;29: 327–339. 10.1038/emboj.2009.358 19959992 PMC2824468

[28] Sycuro LK, Pincus Z, Gutierrez KD, Biboy J, Stern CA, Vollmer W, et al Peptidoglycan crosslinking relaxation promotes *Helicobacter pylori*'s helical shape and stomach colonization. Cell. 2010;141: 822–833. 10.1016/j.cell.2010.03.046 20510929 PMC2920535

[29] Koch MK, McHugh CA, Hoiczyk E. BacM, an N-terminally processed bactofilin of *Myxococcus xanthus*, is crucial for proper cell shape. Mol Microbiol. 2011;80: 1031–1051. 10.1111/j.1365-2958.2011.07629.x 21414039 PMC3091990

[30] Bulyha I, Lindow S, Lin L, Bolte K, Wuichet K, Kahnt J, et al Two small GTPases act in concert with the bactofilin cytoskeleton to regulate dynamic bacterial cell polarity. Dev Cell. 2013;25: 119–131. 10.1016/j.devcel.2013.02.017 23583757

[31] Kaiser D. Social gliding is correlated with the presence of pili in *Myxococcus xanthus* . Proc Natl Acad Sci USA. 1979;76: 5952–5956. 42906 10.1073/pnas.76.11.5952 PMC411771

[32] Bosch J, Turley S, Daly TM, Bogh SM, Villasmil ML, Roach CM, et al Structure of the MTIP-MyoA complex, a key component of the malaria parasite invasion motor. Proc Natl Acad Sci USA. 2006;103: 4852–4857. 16547135 10.1073/pnas.0510907103 PMC1458759

[33] Bosch J, Turley S, Roach CM, Daly TM, Bergman LW, Hol WG. The closed MTIP-myosin A-tail complex from the malaria parasite invasion machinery. J Mol Biol. 2007;372: 77–88. 17628590 10.1016/j.jmb.2007.06.016 PMC2702245

[34] Zeth K, Diederichs K, Welte W, Engelhardt H. Crystal structure of Omp32, the anion-selective porin from *Comamonas acidovorans*, in complex with aperiplasmic peptide at 2.1 Å resolution. Structure. 2000;8: 981–992. 10986465 10.1016/s0969-2126(00)00189-1

[35] Xu D, Zhang Y. *Ab initio* protein structure assembly using continuous structure fragments and optimized knowledge-based force field. Proteins. 2012;80: 1715–1735. 10.1002/prot.24065 22411565 PMC3370074

[36] Zhang Y. I-TASSER server for protein 3D structure prediction. BMC Bioinformatics. 2008;9: 40 10.1186/1471-2105-9-40 18215316 PMC2245901

[37] Roy A, Kucukural A, Zhang Y. I-TASSAR: a unified platform for automated protein structure and function prediction. Nat Protoc. 2010;5: 725–738. 10.1038/nprot.2010.5 20360767 PMC2849174

[38] Kim DE, Chivian D, Baker D. Protein structure and analysis using the Robetta Server. Nucleic Acids Res. 2004;32: W526–W531. 15215442 10.1093/nar/gkh468 PMC441606

[39] Zhang J, Wang Q, Barz B, He Z, Kosztin I, Shang Y, et al MUFOLD: a new solution for protein 3D structure prediction. Proteins. 2010;78: 1137–1152. 10.1002/prot.22634 19927325 PMC2885889

[40] Cheng J. A multi-template combination algorithm for protein comparative modeling. BMC Struct Biol. 2008;8: 18 10.1186/1472-6807-8-18 18366648 PMC2311309

[41] Wang Z, Eickholt J, Cheng J. MULTICOM: A multi-level combination approach to protein structure prediction and its assessment in CASP8. Bioinformatics. 2010;26: 882–888. 10.1093/bioinformatics/btq058 20150411 PMC2844995

[42] Kelley LA, Sternberg MJE. Protein structure prediction on the Web: a case study using the Phyre server. Nat Protoc. 2009;4: 363–371. 10.1038/nprot.2009.2 19247286

[43] Kozakov D, Beglov D, Bohnuud T, Mottarella SE, Xia B, Hall DR, et al How good is automated protein docking? Proteins. 2013;81: 2159–2166. 10.1002/prot.24403 23996272 PMC3934018

[44] Krissinel E, Henrick K. Inference of macromolecular assemblies from crystalline state. J Mol Biol. 2007;372: 774–797. 17681537 10.1016/j.jmb.2007.05.022

[45] Greenfield NJ. Using circular dichroism to estimate protein secondary structure. Nat Protoc. 2006;1: 2876–2890. 17406547 10.1038/nprot.2006.202 PMC2728378

[46] Arrondo JLR, Muga A, Castesana J, Goñi FM. Quantitative studies of the structure of proteins in solution by Fourier-transform infrared spectroscopy. Prog Biophys Mol Biol. 1993;59: 23–56. 8419985 10.1016/0079-6107(93)90006-6

[47] Barth A, Zscherp C. What vibrations tell us about proteins. Quarterly Rev Biophys. 2002;35: 369–430. 12621861 10.1017/s0033583502003815

[48] Zandomeneghi G, Krebs MRH, McCammon MG, Fändrich M. FTIR reveals structural differences between native β-sheet proteins and amyloid fibrils. Prot Sci. 2004;13: 3314–3321. 15537750 10.1110/ps.041024904 PMC2287307

[49] Huang YJ, Mao B, Aramini JM, Montelione GT. Assessment of template-based protein structure predictions in CASP10. Proteins. 2014;82: 43–56. 10.1002/prot.24488 24323734 PMC3932189

[50] Yeh TY, Kowalska AK, Scipioni BR, Cheong FKY, Zheng M, Derewenda U, et al Dynactin helps target Polo-like kinase 1 to kinetochores via its left-handed beta-helical p27 subunit. EMBO J. 2013;32: 1023–1035. 10.1038/emboj.2013.30 23455152 PMC3616283

[51] Biegert A, Söding J. HHrepID: de novo protein repeat identification by probabilistic consistency. Bioinformatics. 2008;24: 807–814. 10.1093/bioinformatics/btn039 18245125

[52] Crooks GE, Hon G, Chandonia JM, Brenner SE. WebLogo: a sequence logo generator. Genome Res. 2004;14: 1188–1190. 15173120 10.1101/gr.849004 PMC419797

[53] Schneider TD, Stephens RM. Sequence logos: a new way to display consensus sequences. Nucleic Acids Res. 1990;18: 6097–6100. 2172928 10.1093/nar/18.20.6097 PMC332411

[54] Ruzin SE. Plant Microtechnique and Microscopy. Oxford University Press; 1999.

[55] Ojeda-Lopez MA, Needleman DJ, Song C, Ginsburg A, Kohl PA, Li Y, et al Transformation of taxol-stabilized microtubules into inverted tubulin tubules triggered by a tubulin conformation switch. Nat Mater. 2014;13: 195–203. 10.1038/nmat3858 24441880 PMC3946914

[56] Löwe J, Amos LA. Crystal structure of the bacterial cell-division protein FtsZ. Nature. 1998;391: 203–206. 9428770 10.1038/34472

[57] Zheng J, Zanuy D, Haspel N, Tsai CJ, Alemán C, Nussinov R. Nanostructure design using protein building blocks enhanced by conformationally constrained synthetic residues. Biochem. 2007;46: 1205–1218. 17260950 10.1021/bi061674a

[58] Jenkins J, Pickersgill R. The architecture of parallel beta-helices and related folds. Prog Biophys Mol Biol. 2001;77: 111–175. 11747907 10.1016/s0079-6107(01)00013-x

[59] Iengar P, Joshim NV, Balaram P. Conformational and sequence signatures in beta helix proteins. Structure. 2006;14: 529–542. 16531237 10.1016/j.str.2005.11.021

[60] Kajava AV, Steven AC. Beta-rolls, beta-helices, and other beta-solenoid proteins. Adv Protein Chem. 2006;73: 55–96. 17190611 10.1016/S0065-3233(06)73003-0

[61] Gauthier SY, Kay CM, Sykes BD, Walker VK, Davies PL. Disulfide bond mapping and structural characterization of spruce budworm antifreeze protein. Eur J Biochem. 1998;258: 445–453. 9874210 10.1046/j.1432-1327.1998.2580445.x

[62] Schuler B, Rachel R, Seckler R. Formation of fibrous aggregates from a non-native intermediate: the isolated P22 tailspike beta-helix domain. J Biol Chem. 1999;274: 18589–18596. 10373469 10.1074/jbc.274.26.18589

[63] Bryan AWJr, Starner-Kreinbrink JL, Hosur R, Clark PL, Berger B. Structure-based predictions reveals capping motifs that prevent β-helix aggregation. Proc Natl Acad Sci USA. 2011;108: 11099–11104. 10.1073/pnas.1017504108 21685332 PMC3131356

[64] Nogales E, Wang H-W, Niederstrasser H. Tubulin rings: which way do they curve? Curr Opin Struct Biol. 2003;13: 256–261. 12727521 10.1016/s0959-440x(03)00029-0

[65] Williamson MP. The structure and function of proline-rich regions in proteins. Biochem J. 1994;297: 249–260. 8297327 10.1042/bj2970249 PMC1137821

[66] Rath A, Davidson AR, Deber CM. The structure of “unstructured” regions in peptides and proteins: role of the polyproline II helix in protein folding and recognition. Biopolymers. 2005;80: 179–185. 15700296 10.1002/bip.20227

[67] Freund C, Schmalz HG, Sticht J, Kühne R. Proline-rich sequence recognition domains (PRD): ligands, function and inhibition. Handb Exp Pharmacol. 2008;186: 407–429. 10.1007/978-3-540-72843-6_17 18491062

[68] Nogales E, Downing KH, Amos A, Löwe J. Tubulin and FtsZ form a distinct family of GTPases. Nat Struct Biol. 1998;5: 451–458. 9628483 10.1038/nsb0698-451

[69] Govaerts C, Wille H, Prusiner SB, Cohen FE. Evidence for assembly of prions with left-handed beta-helices into trimers. Proc Natl Acad Sci USA. 2004;101: 8342–8347. 15155909 10.1073/pnas.0402254101 PMC420396

[70] Stork M, Giese A, Kretzschmar HA, Tavan P. Molecular dynamics simulations indicate a possible role of parallel beta-helices in seeded aggregation of poly-Gln. Biophys J. 2005;88: 2442–2451. 15665127 10.1529/biophysj.104.052415 PMC1305343

[71] Langedijk JP, Fuentes G, Boshuizen R, Bonvin AM. Two-rung model of a left-handed beta-helix for prions explains species barriers and strain variation in transmissible spongiform encephalopathies. J Mol Biol. 2006;360: 907–920. 16782127 10.1016/j.jmb.2006.05.042

[72] Merlino A, Esposito L, Vitagliano L. Polyglutamine repeats and beta-helix structure: molecular dynamics structure. Proteins. 2006;63: 918–927. 16514608 10.1002/prot.20941

[73] Waterhouse AM, Procter JB, Martin DMA, Clamp M, Barton GJ. Jalview version 2—a multiple sequence alignment editor and analysis workbench. Bioinfomatics. 2009;25: 1189–1191. 10.1093/bioinformatics/btp033 19151095 PMC2672624

