# Supplementary figures and images for: The Bactofilin Cytoskeleton Protein BacM of Myxococcus xanthus Forms an Extended β-Sheet Structure Likely Mediated by Hydrophobic Interactions
Source: PLoS One. 2015 Mar 24;10(3):e0121074. doi: 10.1371/journal.pone.0121074 (PMC4372379; doi:10.1371/journal.pone.0121074)

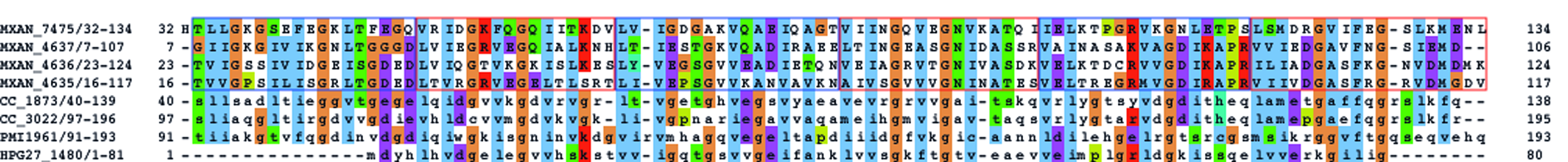

Supplement: S2 Fig — 4 M. xanthus bactofilin paralogs are aligned with BacA and BacB of C. crescentus (CC_1873 and CC_3022, respectively), CcmA from P. mirabilis (PMI1961) and CcmA from H. pylori (HPG27_1480). (TIF) [file pone.0121074.s002.tif]

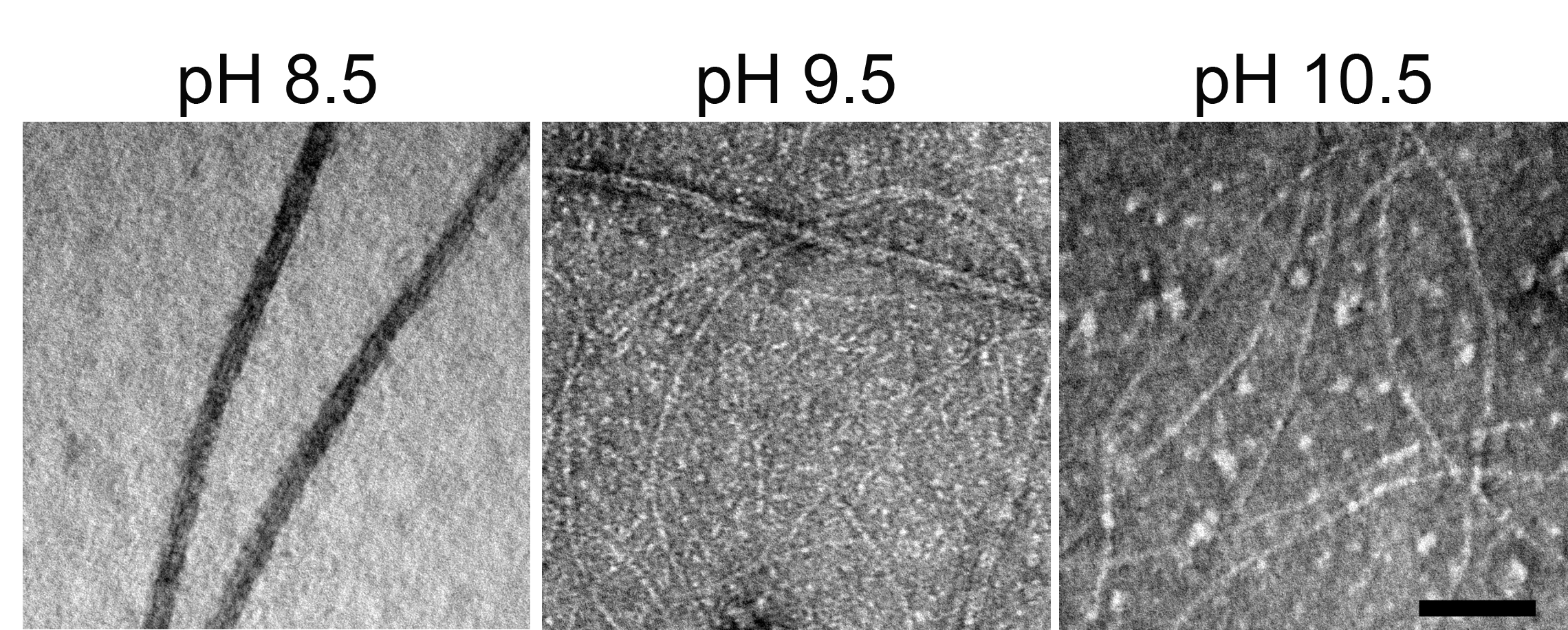

Supplement: S3 Fig — Exogenously expressed and purified BacM polymerizes into different structures upon dialysis against 20 mM glycine buffer at various pH. At pH 8.5 10 nm fibers resembling those isolated from M. xanthus cells predominate (pH 8.5). Upon further increase of the pH, the 10 nm fibers are more and more replaced by 3 nm filaments (pH 9.5), which at pH 10.5 are the only observed form of polymerized BacM (pH 10.5). Scale bar, 50 nm. (TIF) [file pone.0121074.s003.tif]

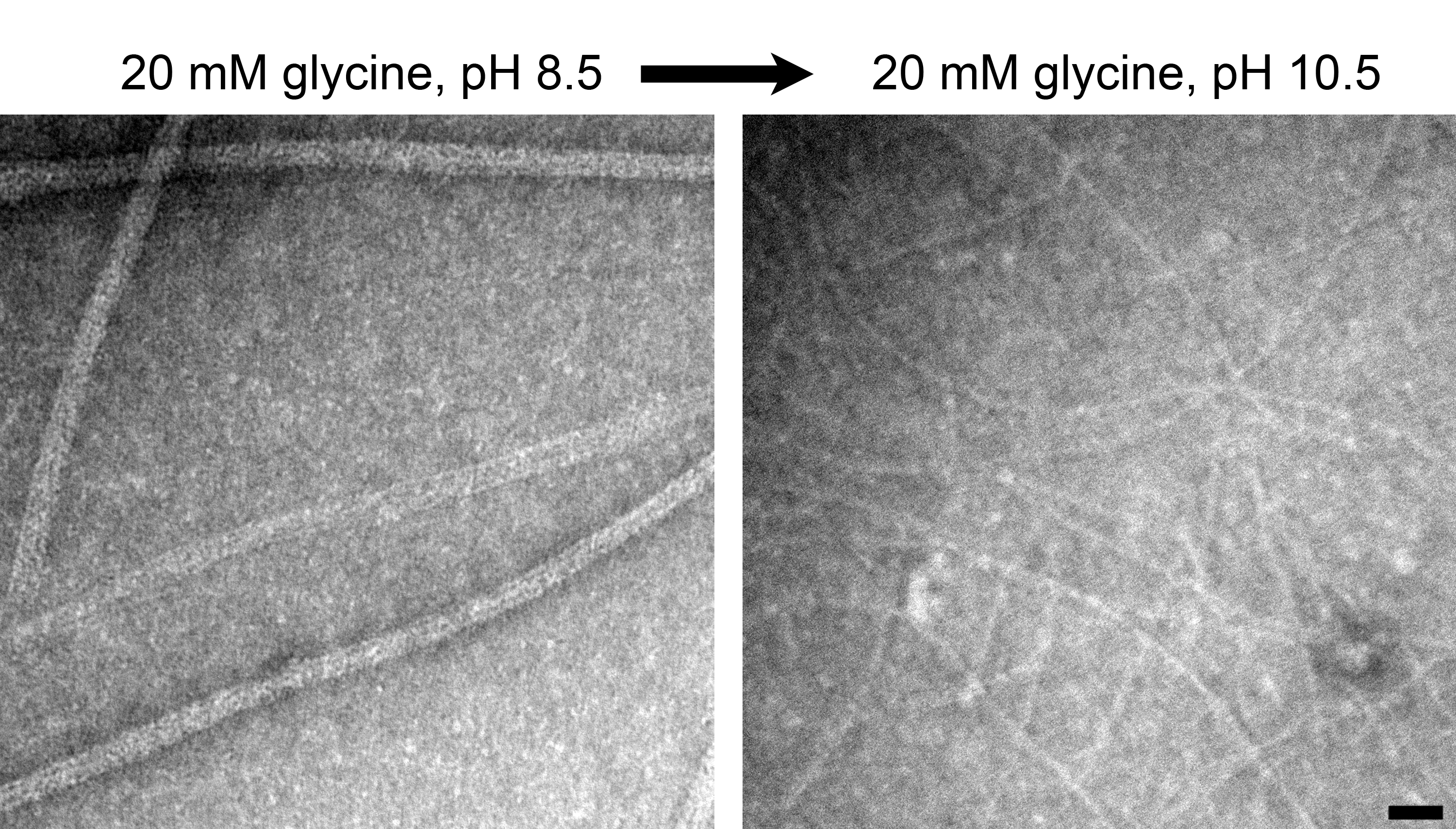

Supplement: S4 Fig — BacM was purified in 8M urea, and subsequently dialyzed against 20 mM glycine, pH 8.5. A sample was removed, applied to a copper grid, negative stained, and imaged by transmission electron microscope (left). The remainder of the dialysate was then dialyzed against 20 mM glycine, pH 10.5 and imaged as above (right). Scale bar = 25 nm. (TIF) [file pone.0121074.s004.tif]

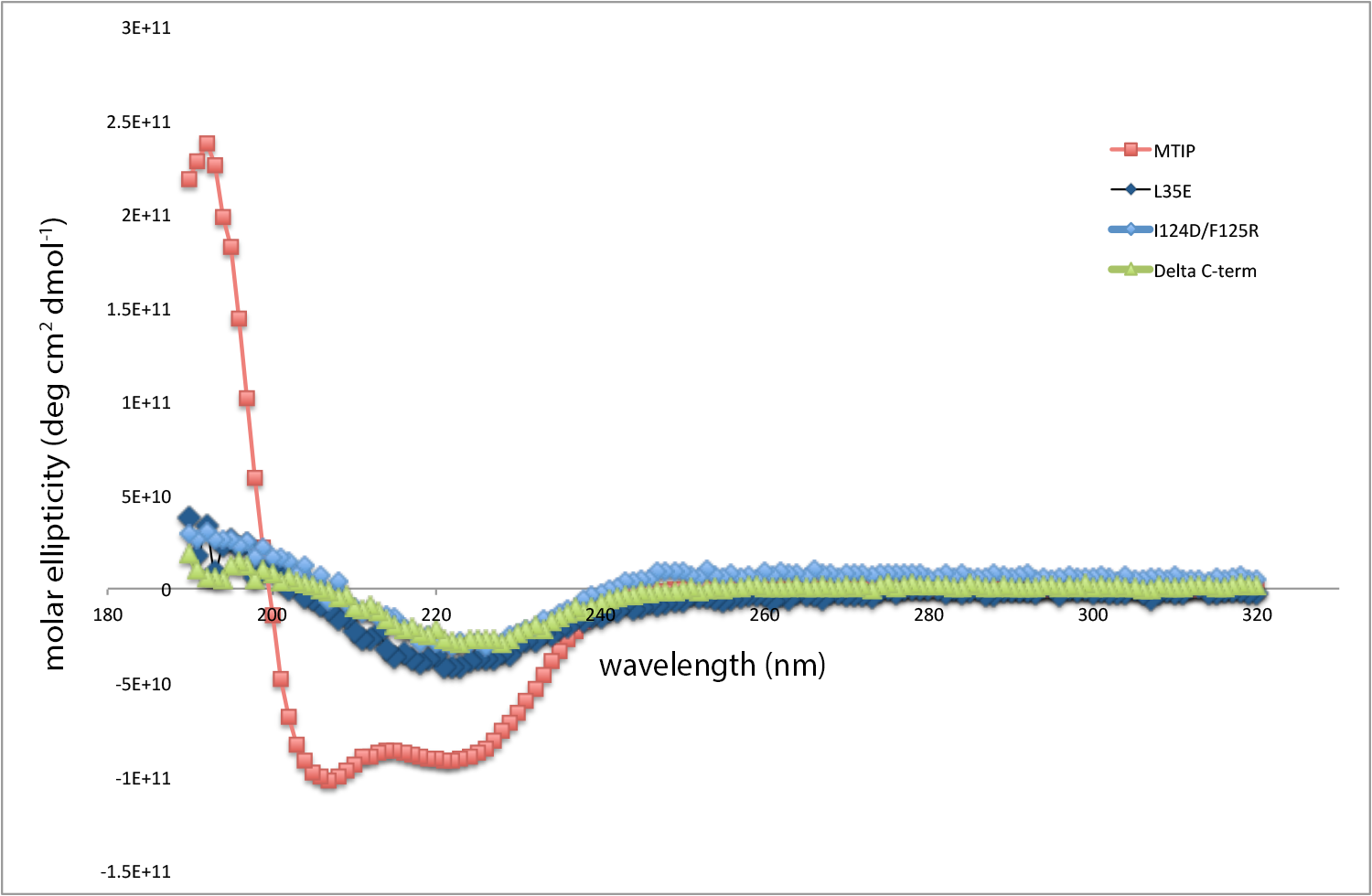

Supplement: S5 Fig — The indicated BacM mutants were purified as described in Materials and Methods, and dialyzed against 20 mM Tris, pH 7.5 to a final concentration of 0.5–1.0 mg/ml. These dialysates were examined by circular dichroism spectroscopy, as described in Materials and Methods. The α–helix-only protein MTIP was used as a control [32,33]. (TIF) [file pone.0121074.s005.tif]

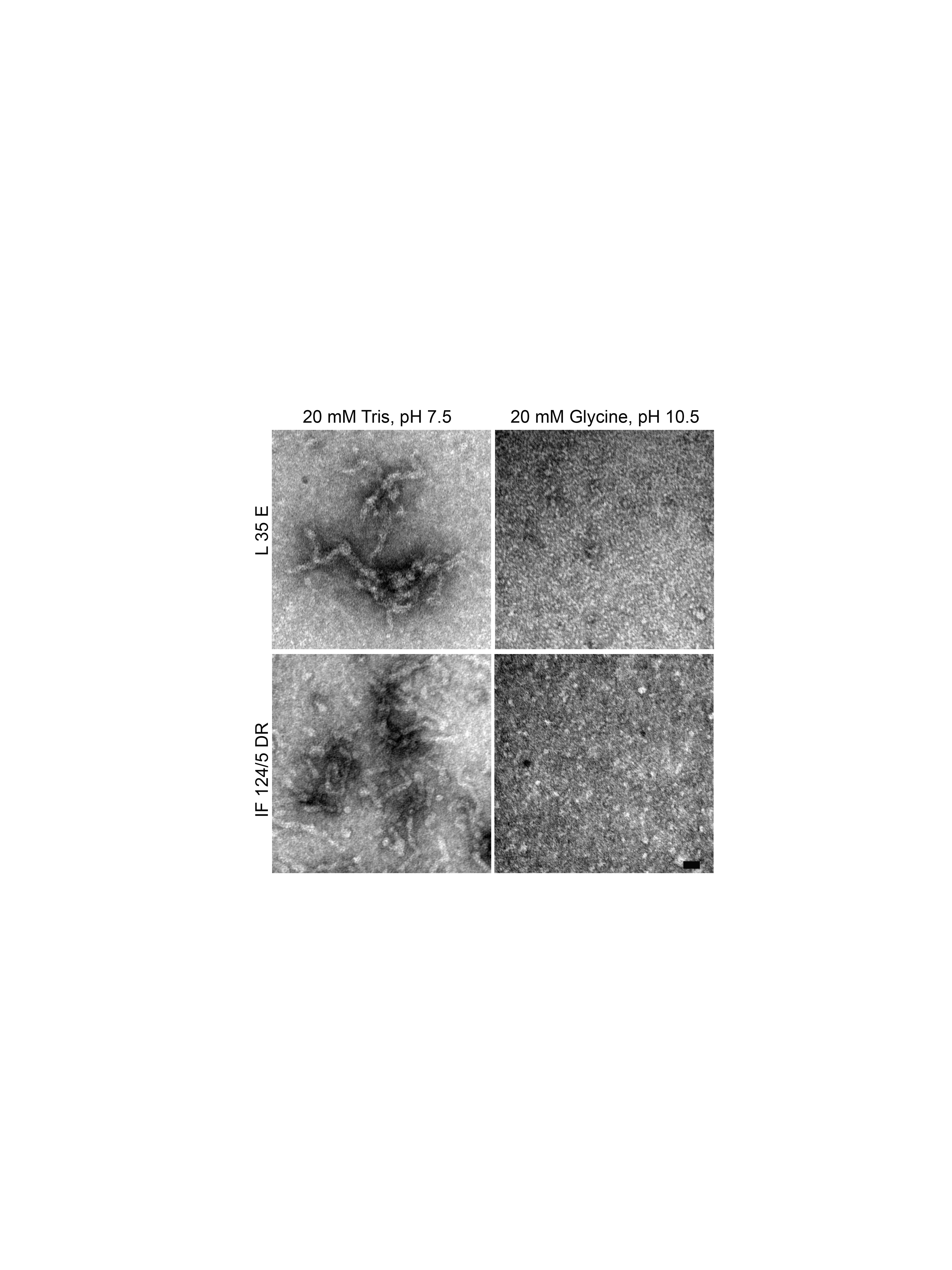

Supplement: S6 Fig — The indicated mutants of BacM were expressed in E. coli and purified in 8 M urea, and subsequently dialyzed against 20 mM Tris, pH 7.5 (left) or 20 mM glycine, pH 10.5 (right). Samples were applied to a copper grid, negative stained, and imaged by transmission electron microscope. While aggregates were ubiquitously found at pH 7.5, they were absent in samples prepared from the glycine buffer. Scale bar = 25 nm. (TIF) [file pone.0121074.s006.tif]

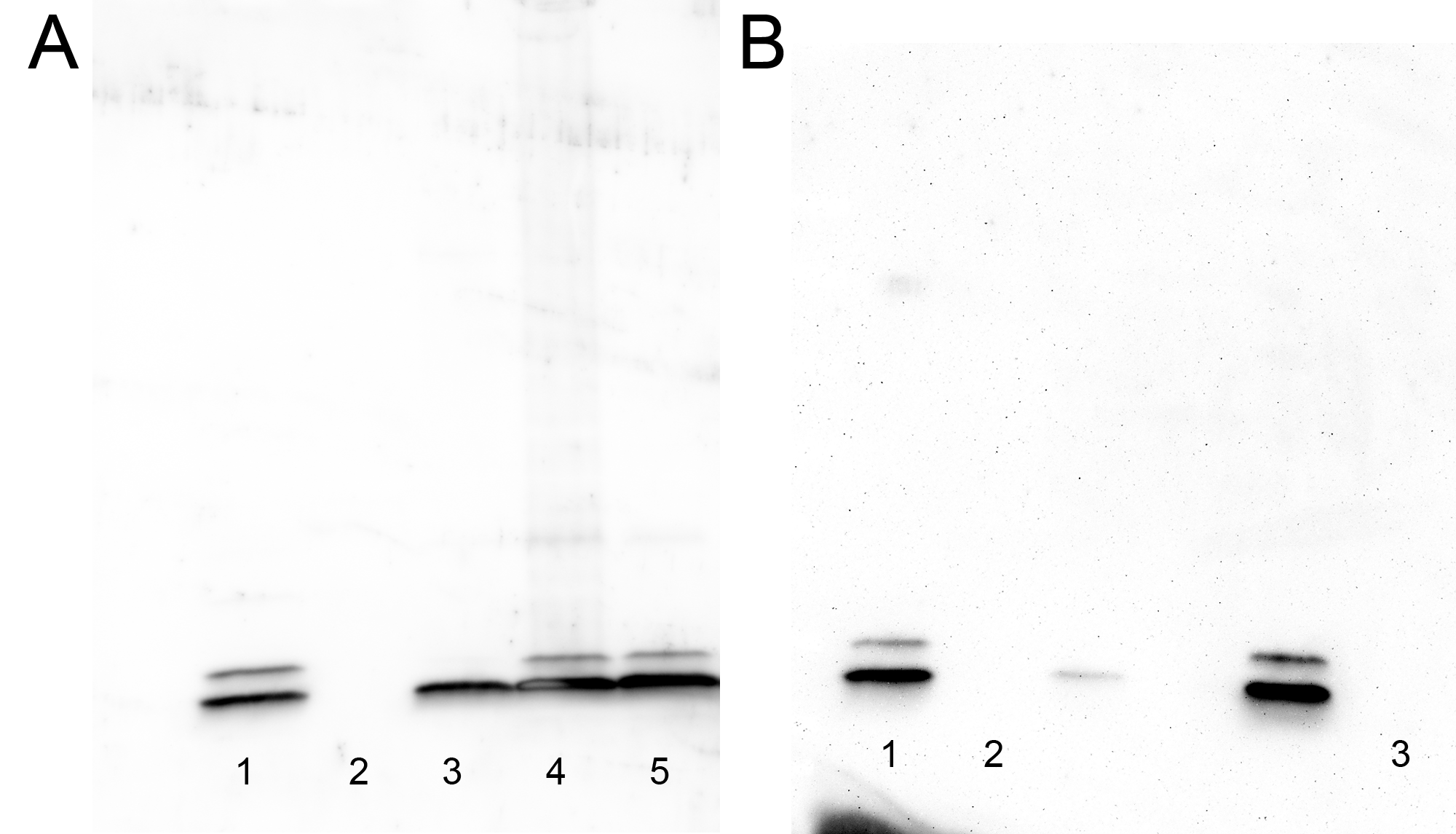

Supplement: S7 Fig — Lysates from the indicated strains of M. xanthus were separated by SDS-PAGE and immunoblotted with an affinity purified anti-BacM antibody [29]. (A) Lane 1: wildtype (DK1622); Lane 2: ΔbacM (EH301); Lane 3 ΔbacM with wildtype bacM rescue (EH344); Lane 4: ΔbacM with I124D/F125R bacM rescue (EH171); Lane 5: ΔbacM with L35E bacM rescue (EH175). (B) Lane 1: wildtype (DK1622); Lane 2: ΔbacM (EH301); Lane 3 ΔbacM with bacM-ΔC-term rescue (EH106). Unlabeled lanes are from strains not discussed in this report. (TIF) [file pone.0121074.s007.tif]
