# Supplementary material for: The Bactofilin Cytoskeleton Protein BacM of Myxococcus xanthus Forms an Extended β-Sheet Structure Likely Mediated by Hydrophobic Interactions
Source: PLoS One. 2015 Mar 24;10(3):e0121074. doi: 10.1371/journal.pone.0121074 (PMC4372379; doi:10.1371/journal.pone.0121074)
Supplement: S1 Fig — The 30 structures used as templates to generate homology models by five servers were aligned using ClustalW. The sequences have been aligned with the BacM sequence residues 28–150. ClustalX coloring was applied to sequences in Jalview to denote amino acid conservation [73]. (PDF) [file pone.0121074.s001.pdf]

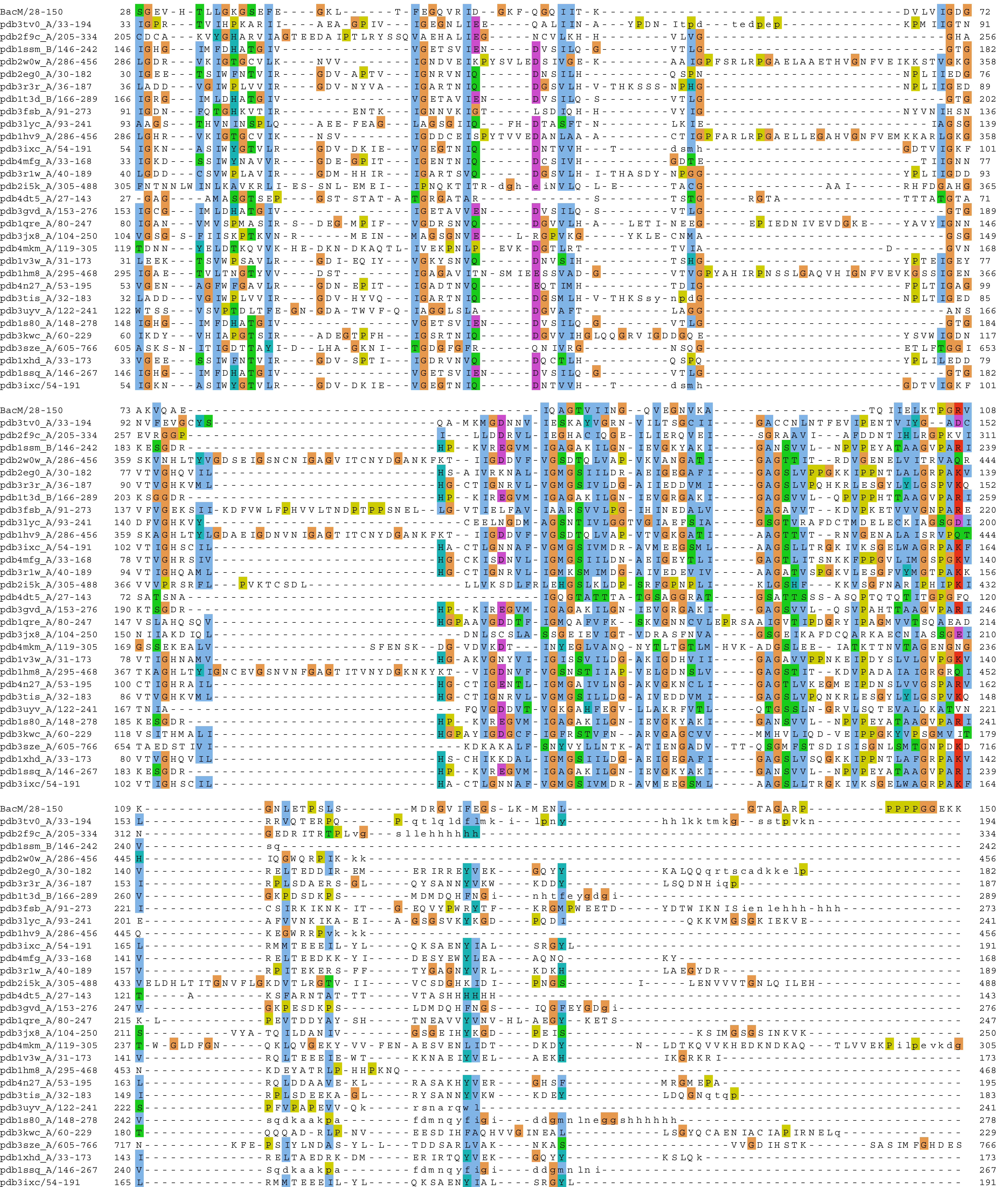

BacM/28-150
pdb3tv0_A/33-194
pdb2f9c_A/205-334
pdb1ssm_B/146-242
pdb2w0w_A/286-456
pdb2eg0_A/30-182
pdb3r3r_A/36-187
pdb1t3d_B/166-289
pdb3fsb_A/91-273
pdb3lyc_A/93-241
pdb1hv9_A/286-456
pdb3ixc_A/54-191
pdb4mfg_A/33-168
pdb3r1w_A/40-189
pdb2i5k_A/305-488
pdb4dt5_A/27-143
pdb3gvd_A/153-276
pdb1qre_A/80-247
pdb3jx8_A/104-250
pdb4mkm_A/119-305
pdb1v3w_A/31-173
pdb1hm8_A/295-468
pdb4n27_A/53-195
pdb3tis_A/32-183
pdb3uyv_A/122-241
pdb1s80_A/148-278
pdb3kwc_A/60-229
pdb3sze_A/605-766
pdb1xhd_A/33-173
pdb1ssq_A/146-267
pdb3ixc/54-191
